# Recombinant SpTransformer proteins bind to specific sites on sea urchin phagocytes and modulate *SpTransformer* gene expression and immune responsiveness

**DOI:** 10.3389/fimmu.2024.1496832

**Published:** 2025-01-28

**Authors:** Ryley S. Crow, Leon Grayfer, L. Courtney Smith

**Affiliations:** Department of Biological Sciences, George Washington University, Washington, DC, United States

**Keywords:** echinoderm, *Strongylocentrotus purpuratus*, coelomocytes, innate immunity, immune effector proteins, auto-regulation

## Abstract

**Introduction:**

The California purple sea urchin, *Strongylocentrotus purpuratus*, relies exclusively on an innate immune system to survive in its pathogen rich marine environment. Central to this defense is the *SpTransformer (SpTrf)* gene family that is unique to the euechinoid group of echinoderms. These genes were initially identified based on their striking upregulation in response to immune challenge. The *SpTrf* gene family encodes structurally similar proteins with a wide range of sequence diversity within and among individual sea urchins. A recombinant (r)SpTrf protein interacts specifically with a variety of non-self targets. Other rSpTrf proteins cross-linked to inert beads show distinct functions for cell binding and augmenting phagocytosis . However, whether the rSpTrf proteins bind to sea urchin phagocytes, and the cellular consequences of binding are largely unexplored.

**Methods:**

rSpTrf protein binding to, and responses by phagocytes was investigated by cytology, flow cytometry, binding competitions using In-cell ELISA, and gene expression analyses.

**Results:**

Soluble rSpTrf proteins bind specifically and exclusively to both live and fixed polygonal and small phagocytes. The different rSpTrf proteins appear to bind shared receptor(s) or other form of cell surface binding site. The phagocyte response to bound rSpTrf proteins culminates in modulated expression of the *SpTrf* gene family as well as other immune-related genes.

**Conclusions:**

These findings underscore the multifaceted and dynamic functions of SpTrf proteins within the innate immune system of the purple sea urchin. Their varied functions enable a robust immune response while also providing a unique modulatory mechanism by which response levels are controlled and adjusted to the level of the foreign threat.

## 1 Introduction

Invertebrates have sophisticated innate immune systems that are finely tuned to distinguish between self and non-self as exemplified by effective host protection against infection through recognition and clearance of foreign targets [*e.g.*, (1–4)]. Genes encoding innate immune proteins in invertebrates include several types of pathogen recognition receptors (PRRs) and a wide variety of immune effector molecules. In response to pathogen pressure, many immune gene families have arisen and expanded through duplications of genes and genomic segments harboring multiple genes, nonsynonymous substitutions within coding regions of genes, and editing of messages, among other mechanisms that result in significant sequence diversity (5–9). Examples include genes that encode the fibrinogen-related proteins in molluscs (10–12), the complement-like C1q family in oysters (13, 14), the variable region-containing chitin-binding proteins in ascidians (15), a variety of antimicrobial peptides in a wide range of marine invertebrates [*e.g.*, (16–19)], Toll-like receptors and nucleotide oligomerization domain-containing (NOD)-like receptors in invertebrates [(20–26), reviewed in (27)], and Transformer (Trf) proteins in euechinoids (28–30); reviewed in (31)).

**Figure 1:**
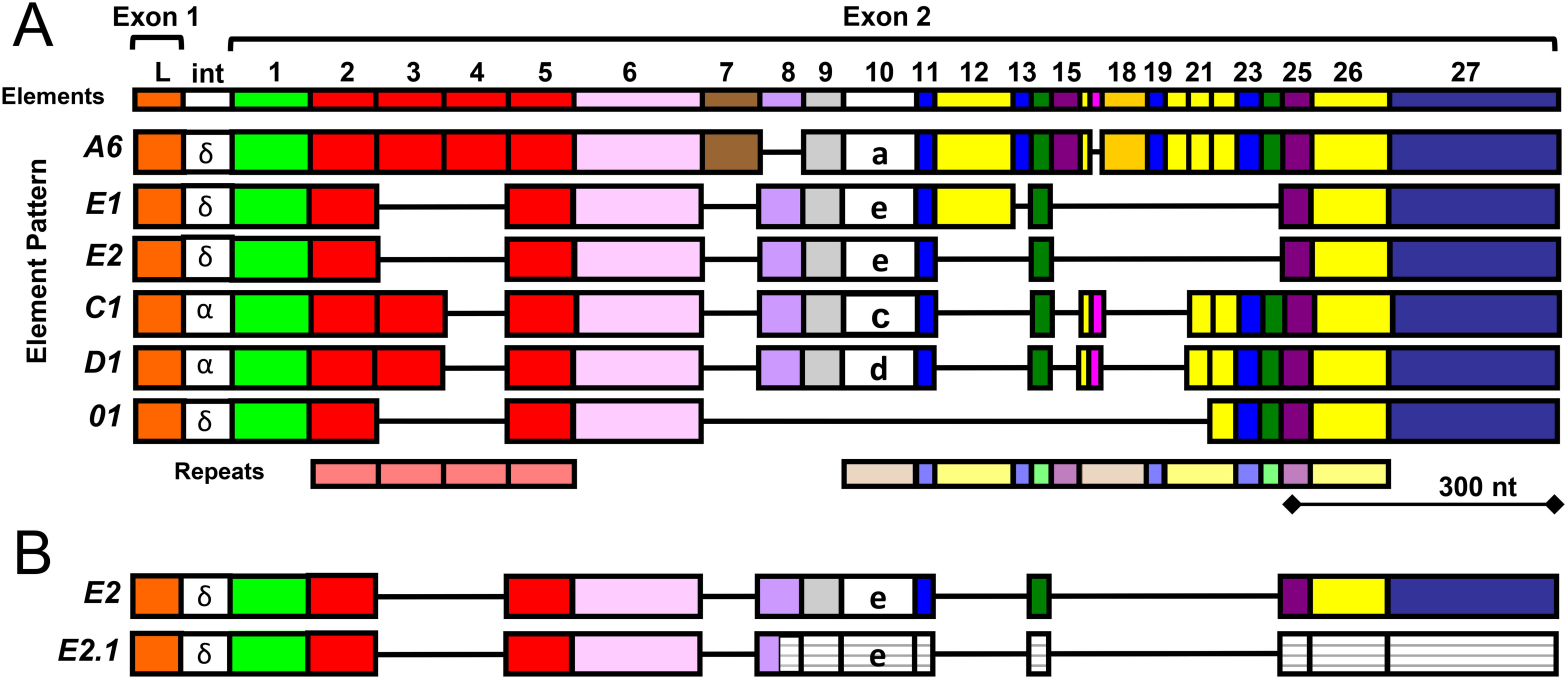
A cartoon alignment shows element patterns in exon 2 of the *SpTrf* genes. **(A)** Gene names, shown to the left, are based on the sequence of element 10, indicated in lower case letters, which is associated with distinct patterns of the surrounding elements. The maximum number of elements (colored boxes) from all known *SpTrf* genes and cDNAs from *S. purpuratus* (28, 37, 38) are shown at the top. Tandem and interspersed repeats in the second exon are shown at the bottom. The intron (int) for most genes is about 400 nucleotides (nt) and is not shown to scale. The intron labels are based on a phylogenetic analysis of introns to establish clades of similar sequence (28). This figure is modified from (39). **(B)** Many *SpTrf-E2* messages are edited to *SpTrf-E2.1*. Most of the *SpTrf* messages are edited that alters the sequence, which expands the range of proteins encoded by individual genes (39). Many of the *SpTrf-E2* messages are edited at a specific glycine codon to create a stop in element 8 that encodes a truncated SpTrf-E2.1 protein (38). The message is not degraded in sea urchin cells because the SpTrf-E2.1 protein is present in the coelomic fluid (43). The white striped elements in the second exon of the *SpTrf-E2.1* message are 3′ of the edited stop codon and are not transcribed.

The *SpTrf* system was first identified in the California purple sea urchin, *Strongylocentrotus purpuratus*, based on striking increases in gene expression in response to immune challenge (32, 33). This gene family shows extraordinary sequence diversity and is estimated to have approximately 50 genes (34) that are clustered in the *S. purpuratus* genome in two known loci (9, 35) and likely within additional unidentified clusters. The gene clusters are associated with a wide range of repeats, consistent with local genomic instability, which is proposed to drive sequence diversity among the genes as a mechanism to keep pace with the rapid evolution of pathogens (8). The second exons in the *SpTrf* genes have multiple recognizable blocks of sequences known as elements that are defined by the insertion of gaps to optimize alignments (**Figure 1A**) (28, 36). There are 27 known elements that have been identified from cDNA and gene sequences, yet no gene includes all elements (28, 37). Elements are often shared among genes, but genes with identical sequences are not shared among animals (34). Element 10 has different sequences that are associated with relatively consistent element patterns in the gene (37). Therefore the sequence of element 10 is used to define genes by name (**Figure 1A**). Individual *Trf* genes identified from several sea urchin species also show distinctive mosaic patterns of the elements in the second exon of the genes (28–30). A mosaic pattern of elements appears to be a common characteristic of the *Trf* system, although there are species specific *Trf* gene families based on variations in sequences and types of elements (29, 30).

The sequence diversity of *SpTrf* genes and messages has been used to deduce the diversity of the encoded proteins that show a predicted size range of 14 to 54 kilodaltons (kDa) (28, 38). The sizes of mature proteins are based on the number and sizes of the elements encoded in the second exon. For example, the deduced protein from the *SpTrf-A6* gene is the longest, whereas the protein encoded by the *SpTrf-01* gene is the shortest and is missing a number of central elements in the second exon including element 10 that defines the gene name (**Figure 1A**). The *SpTrf* messages are edited (39), which diversifies the mRNA sequences derived from individual genes, thereby expanding the protein sequence diversity. Much of the editing, however, is directed at the *SpTrf-E2* messages, changing a glycine codon to a stop in element 8, which deletes about half of the protein (**Figure 1B**) (38). Although SpTrf-E2.1 is truncated, it is expressed by sea urchin cells and has been detected in the coelomic fluid (40). Despite their sequence diversity, the deduced SpTrf protein sequences exhibit an overall conserved structure of a hydrophobic α helical N-terminal leader encoded by exon 1, followed by a glycine (Gly)-rich region (elements 1-5), a multimerization region with a single arginine-glycine-aspartic acid (RGD) integrin binding motif (element 6), a histidine (His)-rich region (elements 7-25), and a C terminal region with up to four possible positions for the stop codon (elements 26-27) (28, 35, 38, 41). This general structure is similar to Trf proteins in other euechinoids (29, 30). Native (nat)Trf proteins in the coelomic fluid (CF) multimerize with one another (29, 30, 42), which is based on a recombinant peptide of the multimerization region (41). The diversity of the Trf proteins has been estimated to be hundreds of slightly different SpTrf proteins that are secreted into the CF in individual sea urchins (43).

Single *SpTrf* genes are expressed in individual cells of the phagocyte class of coelomocytes (44). The natSpTrf proteins are localized in perinuclear vesicles in small and large phagocytes that are consistent with transport vesicles and are also positioned on the surface of small phagocytes (42, 45). The natSpTrf proteins bind to Gram positive and Gram negative bacteria, and when bound to the marine microbe, *Vibrio diazotrophicus*, they augment phagocytosis (41, 46). Because individual natSpTrf proteins cannot be isolated from CF, constructs of select SpTrf proteins were transformed into *E.coli*, but only the rSpTrf-E1 protein (rSpTrf-E1-Ec) was produced successfully, suggesting that expression from the other constructs was lethal to the bacterial cells (41). The rSpTrf-E1-Ec size is consistent with the deduced prediction from the mRNA, it binds a range of targets including Gram negative bacteria and associated pathogen associated molecular patterns (PAMPs), *Saccharomyces cerevisiae* and β-1,3-glucan, but fails to bind Gram positive bacteria and associated PAMPs (41). This protein is unstable, intrinsically disordered, and transforms to α helical upon binding targets such as lipopolysaccharide (LPS) (47). Although it binds to *V. diazotrophicus*, a marine microbe, it does not augment phagocytosis by sea urchin phagocytes (46). Furthermore, the inability of rSpTrf-E1-Ec to augment phagocytosis of a microbial target, unlike a mixture of natSpTrf proteins, suggests that individual native proteins have distinct functions and that they may interact to facilitate a highly effective and multifaceted immune response [(46), reviewed in (31)].

Based on the range of specific targets bound by rSpTrf-E1-Ec, other individual natSpTrf proteins may also have unique repertoires for binding foreign targets. Consequently, seven other rSpTrf proteins with distinct element patterns were expressed in insect cells. They are stable and are modified with N-linked oligosaccharides, which is unlike rSpTrf-E1-Ec (41, 48). When the rSpTrf proteins are cross-linked to inert beads, they show variations in binding to the surface of sea urchin phagocytes and abilities to drive phagocytosis (48). The sequences and element patterns of rSpTrf proteins currently do not correlate with specific functions, which prevents predictions about protein binding targets or opsonin capacity based solely on element patterns. This aligns with the proposed complexity of the natSpTrf system in sea urchins in which many proteins function as opsonins and enhance phagocytosis in a subset of phagocytes (48).

The purple sea urchin, *S. purpuratus*, has several types of coelomocyte in the CF that can be distinguished based on morphology and size [reviewed in (49, 50)], and vary in proportions depending on immune challenge (51). A subset of polygonal and small phagocytes express natSpTrf proteins, which are localized to perinuclear vesicles and the small phagocytes also express natSpTrf proteins on their cell surface [(42, 44, 45) reviewed in (49)]. The polygonal and small phagocytes recognize and take up beads cross-linked with rSpTrf proteins, whereas the discoidal phagocytes display non-specific, base-line phagocytosis (48). The medium phagocytes are rarely observed, but increase in numbers when CF is depleted (52). The other types of coelomocytes include red spherule cells that store echinochrome in cytoplasmic vesicles and displays antibacterial activity when released from the cells (53–55). A number of antimicrobial peptides are expressed by coelomocytes and are important for host immune defense (16, 56). Colorless spherule cells respond to immune challenge with increased cell numbers (57) and may have cytotoxic functions (58). The motile vibratile cells have a single flagellum and are thought to be involved in clotting (59, 60). The variety of cells in the CF highlights a complex and refined immune system in sea urchins, with specialized cells for targeted responses to infection and host defense.

Previous work focused on the functions of both natSpTrf and rSpTrf proteins in foreign target recognition (41, 46, 48, 61), however direct interactions between soluble rSpTrf proteins and coelomocytes has not been addressed. Accordingly, results presented here indicate that the different soluble rSpTrf proteins show variations in binding to both small and polygonal phagocytes. Furthermore, the level of cell binding by each rSpTrf protein is consistent with the level of phagocytosis of inert beads to which it is cross-linked (48). Some of the rSpTrf proteins exhibit binding saturation to cells whereas most do not, and cells from different sea urchins show disparate binding of the proteins. Notably, the different rSpTrf proteins compete for specific putative binding site(s) on phagocytes. Furthermore, binding by soluble rSpTrf proteins to phagocytes modulates the expression of the *SpTrf* gene family as well as the *SpIL17-9* gene, which encodes a pro-inflammatory cytokine (62). Together, the results presented here and those reported previously demonstrate multifunctional activities of the SpTrf proteins that are effective immune response proteins by acting as opsonins, but when bound to phagocytes in the absence of foreign targets, they modulate sea urchin immune responses.

## 2 Materials and methods

### 2.1 Immunocytochemistry

#### 2.1.1 Cyto-centrifuged live coelomocytes

CF was withdrawn from sea urchins as described (63) and diluted [1:2] into calcium-free and magnesium-free sea water with ethylenediaminetetraamino acid (EDTA) and 4-(2-hydroxyethyl)-1-perazineethanesulfonic acid (HEPES) (CMFSW-EH; 460 mM NaCl, 10.7 mM KCl, 7.04 mM Na_2_SO_4_, 2.38 mM NaHCO_3_, 70 mM EDTA, 20 mM HEPES pH 7.4 (46)) according to (63). Cells were counted in a TC20 cell counter (Bio-Rad Laboratories), centrifuged at 5000 x *g* for 5 min at 4°C, and resuspended gently into 500 µl of artificial coelomic fluid (aCF; 398 mM NaCl, 50 mM MgCl_2_, 14 mM KCl, 10 mM CaCl_2_, 1.7 mM NaHCO_3_, 25 mM Na_2_SO_4_, pH 7.4 (38)) using a pipettor with a 1 mL pipette tip that was cut with a sterile razor blade to increase bore diameter and reduce cell shearing. rSpTrf-A6 (1 µg, 36.6 nM) was added to the cells or without added protein (controls) and incubated on ice for 1 hour at a 45° angle to reduce cell settling to the tip of the tube. Cells were agitated every 20 min. Cells were centrifuged at 5000 x *g* for 7 min at 4°C, the supernatant was removed, the cells were resuspended into 500 µl of aCF. The cell suspension was loaded into a chimney (18 mm diameter) of a slide holder cytology assembly (Hettich Zentrifugen) and spun onto Shandon Superfrost Plus positively charged microscope slides (ThermoScientific or Epredia) at 18 x *g* for 15 min at 4°C as optimized and reported previously (48). The assemblies were disassembled, excess aCF was tipped off the slide, and the cells were processed for fixing at room temperature (rt) with pre-fix (0.000025% glutaraldehyde) for 5 min, followed by fix (2% formaldehyde, Triton X-100) in AC320 buffer (320 mM sucrose, 75 mM KCl, 2 mM MgCl_2_, 20 mM EGTA, 20 mM Pipes pH 7.4) for 5 min, permeabilized in ice cold methanol, and washed in standard phosphate buffered saline for 5 min according to published methods (42, 48, 63, 64). Cells were blocked at rt for 45 min in PBS with 5% skim milk power (PBSM) followed by incubation with rabbit-anti-V5 linked to DyLight 549 (RαV5-549; 500X dilution in block; Rockland) and mouse-anti-actin (MαActin; 1500X dilution, MP Biomedicals) for 45 min at rt. Cells were washed three times in PBS for 5 min each and incubated for 45 min at rt with the secondary antibodies; goat-anti-rabbit-Ig linked to Alexa Fluor 555 (GαRIg-555; 1000X dilution; Invitrogen) and goat-anti-mouse-Ig linked to Alexa Fluor 488 (GαMIg-488; 6000X dilution; Invitrogen). After washing with PBS, ProLong Gold antifade reagent with 4′,6-diamidino-2-phenylindole (DAPI) (Thermofisher) was added to the cells, and the glass coverslip was sealed with clear nail polish. Cell imaging was done with a confocal LSM 710 inverted microscope (Zeiss), and the associated digital image editing program was used to change or optimize the fluorescent colors.

For some rSpTrf-A6 binding experiments cells were incubated with two rabbit-anti-natSpTrf antibodies (α-66 and α-68, 2000X dilution in block), and rSpTrf-A6 was detected using chicken-anti-V5 (ChαV5; 1000X dilution in block; Bethyl Laboratories) followed by the secondary antibodies; goat-anti-chicken-Ig linked to Alexa Fluor 405 (GαChIg-405; 1500X dilution; Invitrogen), GαRIg-555 (1500X; Invitrogen), and MαActin (1500X dilution; MP Biomedicals) followed by GαMIg-488 (6000X dilution; Invitrogen). Slide mounting and imaging was the same as described above except ProLong Gold antifade reagent without DAPI (Thermofisher) was added to the cells.

#### 2.1.2 Fixed coelomocytes in culture plates

CF was collected and coelomocytes were counted as described (63). Cells (5.5 X 10^4^) were centrifuged at 18 x *g* for 15 min at 4°C into wells of a 96 well plate with glass bottoms (Nunc MicroWell 96-Well Optical-Bottom Plates, ThermoScientific). The supernatant was removed and the cells were fixed with 4% paraformaldehyde (Electron Microscopy Sciences) in aCF for 20 min at 14°C. The fixative was removed and the wells were washed 3 times with PBS with 0.5% bovine serum albumin (PBS-BSA) for 3 min each wash. Cells were blocked with PBSM for 45 min at rt, followed by incubation with 500 ng (156 nM) of rSpTrf-E2-4 in 100 µl aCF for 45 min at rt. Cells were washed, permeabilized with 100 µl of ice-cold methanol for 5 min at -20°C, washed with PBS-BSA, and incubated with ChαV5 (1000X dilution in PBSM; Bethyl Laboratories) and MαActin (1500X dilution; MP Biomedicals) for 45 min at rt. Cells were washed and incubated with the secondary antibodies GαChIg-405 (1500X dilution; Invitrogen) and GαMIg-488 (6000X dilution; Invitrogen) for 45 min at rt. Cells were washed, and imaged by confocal microscopy, as described above.

### 2.2 Flow cytometry

CF was collected, coelomocytes were counted as described (63), and 10^6^ cells in 1.5 ml tubes were centrifuged at 5000 x *g* for 7 min at 4°C and resuspended in 500 µl of cold aCF as described above. Cells with rSpTrf-A6 (1 µg; 36.6 nM) or without added protein were incubated on ice for 1 hour. The tubes were positioned horizontally to avoid cells settling at the tip, and were agitated every 20 min. Tubes were centrifuged at 5000 x *g* for 7 min at 4°C, and the cells were resuspended in 500 µl of cold block (3% normal mouse serum, 3% BSA in aCF) and incubated on ice for 45 min with agitation every 15 min. Tubes were centrifuged and the cells were resuspended in cold block (250 µl) that contained mouse-anti-V5-Dylight-488 (MαV5-488; 100X dilution; Invitrogen) and incubated for 45 min on ice with agitation every 15 min. Cells were pelleted and resuspended in 500 µl of cold aCF containing propidium iodide (1 ug/ml). Cells were evaluated by flow cytometry on a BD Celesta Cell Analyzer using a gating protocol described previously (30, 63). Results were evaluated and figures were generated with FlowJo (Becton Dickinson).

A Chi-square test with significance set to *p* < 0.05 was used to determine the statistical differences in the scatter plots for the distribution of cells among the four quadrats from each sea urchin after incubation with and without rSpTrf-A6.

### 2.3 In-cell enzyme linked immunosorbent assay

#### 2.3.1 Protein binding to coelomocytes

CF was collected in CMFSW-EH and coelomocytes were counted as described (63). Cells (5.5 X 10^4^) were aliquoted into triplicate wells of a flat bottom Polystyrene 96 well plate (FisherScientific) and the volume per well was adjusted to 100 µl with CMFSW-EH. Cells were centrifuged at 18 x *g* for 10 min at 4°C (48), the supernatant was removed, and the cells were fixed with 4% paraformaldehyde in aCF for 20 min at 14°C. The fixative was removed and the wells were washed with 150 µl PBS-BSA 3 times for 3 min each wash. Cells were blocked with 200 µl PBSM either over night at 4°C or for 1 hour at rt. Wells were washed and cells from at least three animals were incubated with either one of the rSpTrf proteins or recombinant *Xenopus laevis* colony stimulating factor-1 (rCSF-1) in a range of concentrations (0 nM, 2.5 nM, 5 nM, 10 nM, or 20 nM) for 1 hour at rt. Wells were washed and cells were incubated with 100 µl MαV5-HRP (10000X dilution to detect rSpTrf and 2000X dilution to detect rCSF-1; ThermoFisher) for 1 hour at rt. Wells were washed and incubated with 100 µl 3,3′,5,5′-tetramethylbenzidine (TMB) (ThermoFisher) in the dark for 30 min. The color reaction was stopped with 100 µl 1N HCl and wells were read at OD^450^ in a Synergy HTX plate reader (BioTek).

Statistically significant differences in the level of cell binding among the different proteins was established by fitting a linear regression model (*y = mx* + *b*) to estimate the marginal effect of protein concentration on the change in OD^450^. The marginal trends for each protein were compared to identify statistically significant differences using a Tukey test with significance set to *p* < 0.05.

#### 2.3.2 Verification that coelomocytes bind to wells of plates

CF was collected and cells were spun onto wells of a 96 well plate as described (63). Cells were fixed but not permeabilized, and incubated with the α-71 antibody (40000X dilution in PBSM) for 1 hour at rt followed by the secondary antibody GαRIg-HRP (40000X dilution; Invitrogen) for 1 hour at rt. Color development with TMB was carried out for 30 min in the dark, stopped with 1N HCl, and plates were read at OD^450^.

The correlation between the concentration of extracellular natSpTrf proteins and the level of binding by 20 nM of rSpTrf-A6, -E2-3, or -E1 (in separate wells) was established using Pearson’s correlation test with significance set to *p* < 0.05. At least three animals were evaluated for rSpTrf protein binding and natSpTrf detection.

### 2.4 Binding competition assays using In-cell ELISA

#### 2.4.1 Protein biotinylation

Each rSpTrf protein and BSA were biotinylated using EZ-LinkTM Micro Sulfo-NHS-LC-Biotinylation Kit (ThermoScientific) following the manufacturer instruction. Biotin was reconstituted in PBS and added to the rSpTrf proteins and to BSA at a 50-fold molar excess in 500 µl PBS and incubated with rotation for 45 min at rt. The protein-biotin mixes were loaded onto Zeba Spin Desalting columns (7 kDa molecular weight cut off, ThermoScientific) and spun at 1500 x *g* for 2 min at rt to remove unbound biotin. Biotinylated proteins were stored at -80°C until used.

#### 2.4.2 Self competition

Coelomocytes were spun into the wells of a 96 well plate, fixed, and blocked following the In-cell ELISA protocol described above. Biotinylated rSpTrf proteins (B-rSpTrf, 20 nM) were added to increasing concentrations (20 nM, 40 nM, 60 nM, 80 nM) of un-labeled rSpTrf proteins of the same isoform or with no competitor as the control. Protein mixes were incubated with coelomocytes in triplicate wells for 1 hour at rt, and cells from at least three animals per rSpTrf protein were evaluated for self-competition binding. The wells were washed and coelomocytes were incubated with streptavidin-HRP (1 µg/ml; Invitrogen) for 1 hour at rt. The wells were washed, color was developed with TMB, stopped with HCl, and the level of binding was evaluated at OD^450^ as described above. Results were normalized to the controls that omitted a competitor for each rSpTrf protein.

#### 2.4.3 Cross competition

Coelomocytes from at least three sea urchins were fixed in triplicate wells and incubated for 1 hour at rt with a mixture of B-rSpTrf-E2-4 or B-rSpTrf-01 (20 nM) plus the same protein or each of the different un-labeled rSpTrf proteins (80 nM) or BSA (80 nM) that served as the negative control. Wells were washed and binding was evaluated as above with streptavidin-HRP (1 µg/mL; Invitrogen) and read at OD^450^. Results were normalized to the level of binding when rSpTrf proteins were incubated with BSA.

The levels of binding among the competitors in the cross-competition analyses were compared to the normalized control (BSA) and significant differences were established using Dunnett’s test with significance set to *p* < 0.01.

### 2.5 Quantitative RT-PCR

Coelomocytes were collected in CMFSW-EH and counted as described above, adjusted to 10^6^ cells/500 µl in 1.5 ml tubes, and centrifuged at 5000 x *g* for 7 min at 4°C. The initial level of gene expression was determined from cells that were lysed upon collection in 500 µl of Trizol (Fisher Scientific). The remaining samples from each sea urchin were resuspended as described above in 500 µl of aCF and incubated with each rSpTrf protein (1 µg) or rCSF-1 (1 µg) for 4 hours at 14°C with agitation every 20 min. Cells were pelleted and lysed in 500 µl of Trizol. Cell lysates were either processed immediately or stored at -20°C for later processing. Total RNA was isolated using a Direct-zol RNA Microprep kit (ZymoResearch) following the manufacturer instructions that included DNAse I treatment. Total RNA in RNAse-free water was used for subsequent cDNA synthesis using the qScript cDNA supermix kit (Quantabio) following manufacturer instruction.

Gene expression was quantified with the iTaq Universal Sybr Green Supermix (Bio-Rad Laboratories) following the manufacturer instruction, which used 5 µl of Sybr Green Supermix, 2 µM forward and reverse primers (**Supplementary Table S1**), and 2.5 µl of cDNA. All samples evaluated for each gene were run in duplicate in a 96 well qRT-PCR plate (VWR Scientific). The plate was centrifuged at 4000 x *g* for 2 min and evaluated in a CFX96 Real Time System thermal cycler (Bio-Rad Laboratories) with the following program: 95°C for 5 min, 39 cycles of 95°C for 10 sec, 60°C for 30 sec, 95°C for 5 sec, 65°C for 5 sec. Amplicon melt data was obtained with incremental step increases of 5°C from 65°C to 95°C. Gene expression analyses were performed using the 2^-ΔΔ^ *^Ct^* method (65) and expression was normalized to the house keeping gene, *SpL8*.

Statistically significant differences (*p* < 0.01) in fold changes of immune gene expression after 4 hours of incubation were determined using Dunnett’s test, by comparing the negative control (no added protein) to the other samples.

## 3 Results

### 3.1 rSpTrf proteins bind directly to phagocytes in the absence of foreign particles

**Figure 2:**
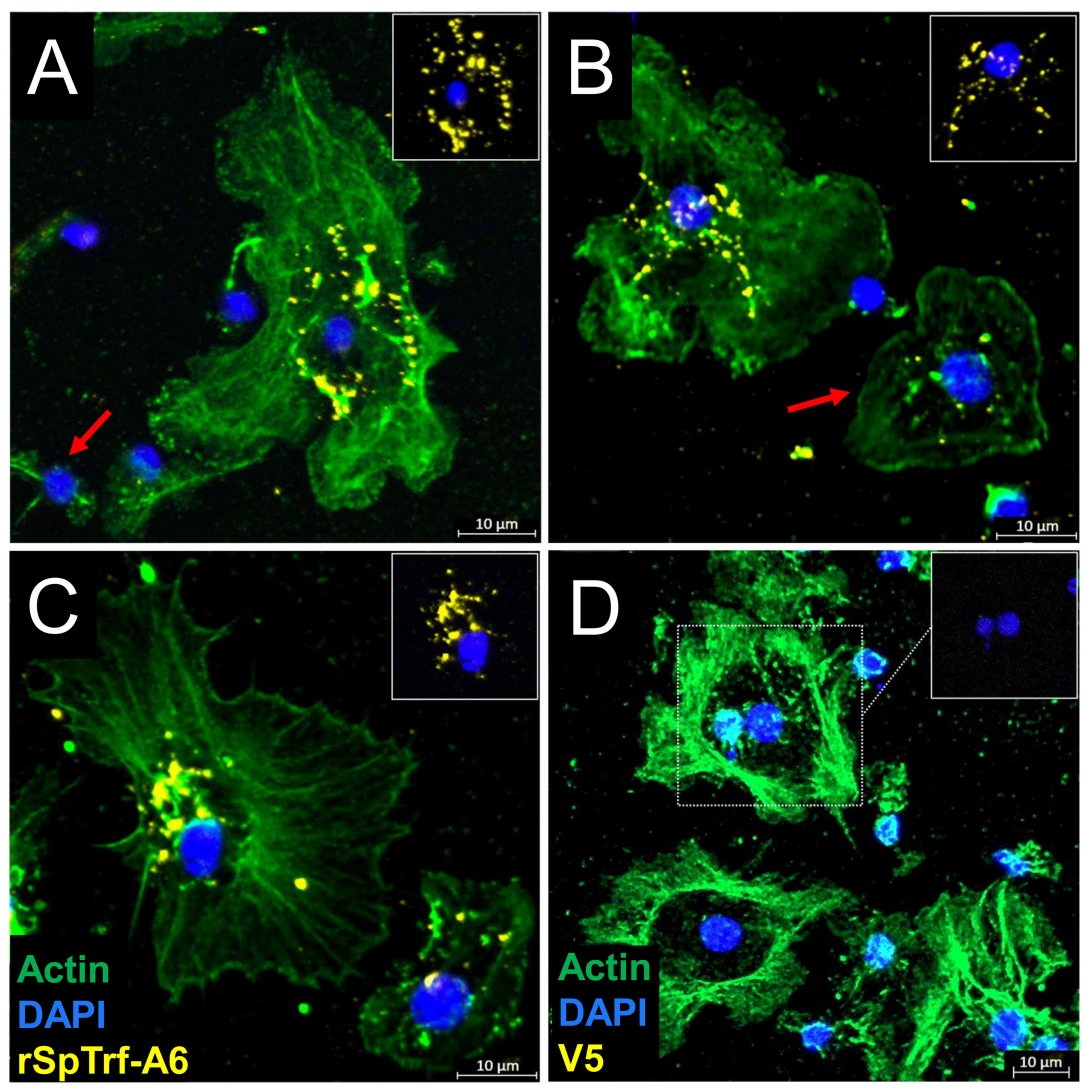
rSpTrf proteins bind to live phagocytes. rSpTrf-A6 binds to a subset of live polygonal phagocytes in variable, punctate, perinuclear patterns. **(A–C)** Phagocytes incubated with rSpTrf-A6 are labeled with RαV5-549 followed by GαRIg-555. Most phagocytes bound rSpTrf-A6, while some showed no binding [red arrow in **(A)**] or very little binding [red arrow in **(B)**]. The arrangement of bound rSpTrf-A6 show patterns that **(A)** surround the nucleus, **(B)** are distributed partially over the nuclear area, or **(C)** are an asymmetrical accumulation on one side of the nucleus. The rSpTrf proteins are generally not associated with the edges of the cells. **(D)** Phagocytes incubated without rSpTrf-A6 are negative for background labeling with RαV5-549 and GαRIg-555. The phagocyte type is identified based on actin cytoskeletal structure, using MαActin followed by GαMIg-488 antibodies. Most cells are polygonal phagocytes, but some small phagocytes with very little actin cytoskeleton associated with the nucleus are present **(A**, **B, D)**. The insets in each panel show the nuclear areas (DNA labeled with DAPI) of the cells with the most rSpTrf-A6 and without the actin label. Imaging was done on an LSM 800 confocal microscope (Zeiss) and false color editing was done with the Zeiss image processing program associated with the microscope. Scale bars indicate 10 µm.

Previous work showed that natSpTrf proteins act as opsonins by binding to *Vibrio diazotrophicus* and enhancing phagocytosis (46). Similarly, when rSpTrf proteins are cross-linked to inert beads, they augment phagocytosis by small and polygonal phagocytes (48). These results suggest that SpTrf proteins interact with designated binding site(s) on coelomocyte surfaces, thus facilitating detection of foreign targets leading to phagocytosis. However, whether the rSpTrf proteins must be associated with a foreign particle for phagocyte recognition remains to be evaluated. To address this question, coelomocytes were incubated in cold aCF with or without soluble rSpTrf-A6. The fixed cells were permeabilized and evaluated on slides with RαV5-549, which recognizes the V5 tag on rSpTrf proteins. The fluorescent signal for rSpTrf-A6 was augmented with a secondary antibody, GαRIg-555, that was conjugated to a fluorophore with the equivalent emission spectrum. MαActin was used to differentiate among the cytoskeletal structures of the different phagocyte types (64, 66, 67). Results showed that soluble rSpTrf-A6 bound to the surface of some phagocytes in a punctate pattern and was generally localized around the nuclear region (**Figure 2**; **Supplementary Figures S1A, B**). This pattern was consistent with the aggregation of rSpTrf-A6 and/or capping and endocytosis of the protein into perinuclear vesicles. While the perinuclear localization of rSpTrf-A6 was observed across phagocytes, there were variations in the overall binding pattern such as encircling the nucleus (**Figure 2A**), a more scattered pattern, including on top of the nucleus (**Figure 2B**), or an asymmetric pattern to one side of the nucleus (**Figure 2C**). Variations in the binding of soluble rSpTrf-A6 aligned with previous reports on phagocyte interactions with rSpTrf-bound targets. Beads cross-linked to rSpTrf proteins bind to the phagocyte surface encircling the nucleus [see Figures 5C, 8B in Crow et al. (48)], while phagocytosed beads cross-linked to rSpTrf proteins or *Vibrio diazotrophicus* opsonized with natSpTrf proteins accumulate in an asymmetrical pattern near one side of the nucleus [see Figure 5C in Crow et al. (48) and Figure 7 in Chou et al. (46)]. Some phagocytes showed very low binding (**Figure 2B**, red arrow)) or no binding to soluble rSpTrf-A6 (**Figure 2A**, red arrow, **Supplementary Figure S1A**, cell 2), consistent with variations in functions among the phagocytes (48). These findings indicated that a subset of live phagocytes were capable of binding soluble rSpTrf-A6 in the absence of interactions with a foreign particle.

**Figure 3:**
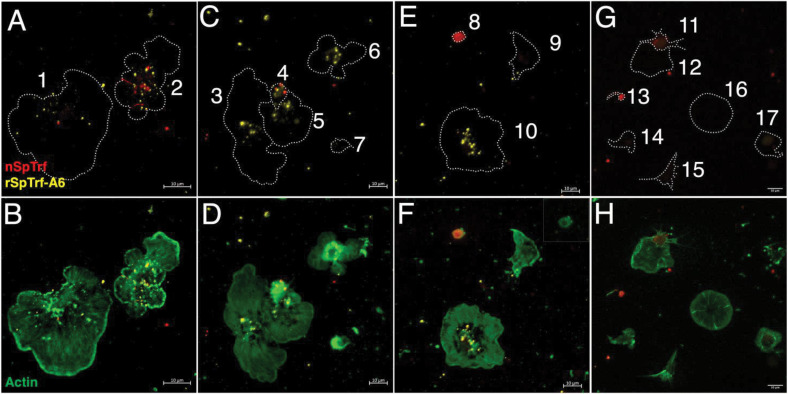
rSpTrf-A6 binds to live phagocytes with variable expression of natSpTrf proteins. Both polygonal and small phagocytes show variable binding of rSpTrf-A6 and variable expression of natSpTrf proteins. **(A–F)** Phagocytes incubated in aCF with rSpTrf-A6 are labeled with ChαV5 followed by GαChIg-405. Cells are also labeled with rabbit-anti-natSpTrf antibodies (α-66 and α-68) followed by GαRIg-555. α-66 and α-68 bind well to natSpTrf proteins but poorly to 36.6 nM rSpTrf-A6. **(G, H)** Phagocytes are incubated as in **(A–F)** but without rSpTrf-A6. All cells are also incubated with MαActin followed by GαMIg-488 and panels in the right column show merges of phagocytes plus actin to identify differences in cytoskeletal structure of different types phagocytes. Nuclear DNA labeling with DAPI is omitted because the emission spectrum overlaps with GαChIg-405. Outlines of cells in the left column of panels are indicated by dotted lines and correlate with the actin staining of the cells in the right column of panels. **(A, B)** Cell 1 binds rSpTrf-A6 (yellow) and shows a single vesicle with natSpTrf proteins. Cell 2 binds rSpTrf-A6 and expresses natSpTrf proteins (red) in a perinuclear pattern. Both cells are polygonal phagocytes. **(C, D)** Cells 3, 5 and 6 are polygonal phagocytes that bind rSpTrf-A6 but do not express natSpTrf proteins. Cells 4 and 7 are small phagocytes and cell 4 binds rSpTrf-A6 and expresses natSpTrf proteins, whereas cell 7 is negative for both. **(E, F)** Cell 8 is a small phagocyte that expresses natSpTrf proteins but does not bind rSpTrf-A6. The inset in **(F)** shows cell 8 with ChαV5 and MαActin labeling without the anti-natSpTrf labeling to confirm the absence of rSpTrf-A6 binding. Cells 9 and 10 are polygonal phagocytes that do not expresses natSpTrf proteins even though cell 10 binds rSpTrf-A6 and cell 9 does not. **(G, H)** Phagocytes incubated without rSpTrf-A6 do not show background labeling for ChαV5 followed by GαChIg-405. However, cells 11 and 13, which are small phagocytes, express natSpTrf proteins. Labeling with α-66 and α-68 followed by GαRIg-555 does not show binding artifacts in the 405 channel. Imaging was done on an LSM 800 confocal microscope (Zeiss) and false color editing for all panels was done with the Zeiss image processing program associated with the microscope. Scale bars indicate 10 µm.

Large and small phagocytes express natSpTrf proteins that are localized to transport vesicles and small phagocytes that also express natSpTrf proteins on their surface (42, 44, 45). It is notable that these are the same types of phagocytes that interact with rSpTrf proteins cross-linked to inert beads (48). To explore the relationship between natSpTrf protein expression and the ability to bind soluble rSpTrf-A6, live phagocytes were incubated with two rabbit-anti-natSpTrf antibodies (α-66 and α-68 show low binding to rSpTrf-A6; unpublished data) to label the natSpTrf proteins, and with ChαV5 antibody to label rSpTrf-A6. Results revealed diversity within each type of phagocyte for the expression of natSpTrf proteins and for binding of rSpTrf-A6 (**Figure 3**). Some polygonal phagocytes bound rSpTrf-A6 and expressed natSpTrf proteins (**Figures 3A, B**, cells 1 and 2), some bound rSpTrf-A6 but did not express natSpTrf proteins (**Figures 3C, D**, cells 3, 5, and 6; **Figure 3E**, cell 10), and some did not bind rSpTrf-A6 or express natSpTrf proteins (**Figure 3E**, cell 9). Notably, there were no polygonal phagocytes that bound rSpTrf-A6 but did not express natSpTrf proteins. Similarly, some small phagocytes bound rSpTrf-A6 and expressed natSpTrf proteins (**Figure 3C**, cell 4). Conversely, some did not bind rSpTrf-A6 but expressed natSpTrf proteins (**Figure 3E**, cell 8), and some did not bind rSpTrf-A6 or express natSpTrf proteins (**Figure 3C**, cell 7). Expression of the natSpTrf proteins by small phagocytes was variable, with either punctate patterns (**Figure 3C**, cell 4) or uniform coating of cell surfaces (**Figure 3E**, cell 8). Low to no background labeling was observed when control cells were incubated in the absence of rSpTrf-A6 followed by incubation with ChαV5 (**Figures 3G**, **H**). For cells incubated with rSpTrf-A6, quantification of rSpTrf-A6 binding and natSpTrf expression was based on fluorescence intensity and showed no clear trends with no difference in average fluorescence between rSpTrf-A6 and natSpTrf detection (**Supplementary Figure S2A**). No notable patterns were observed for individual cells with respect to the magnitude of natSpTrf protein expression relative to the extent of rSpTrf-A6 binding (**Supplementary Figures S2B, C**). However, there tended to be fewer cells (albeit not significantly) that expressed natSpTrf proteins when incubated with rSpTrf-A6 compared to those incubated without added protein (**Supplementary Figure S3**). Overall, these findings suggested that the polygonal and small phagocytes that expressed natSpTrf proteins could also bind soluble rSpTrf proteins and that binding variability for the rSpTrf proteins was evident among cells of both types of phagocytes.

**Figure 4:**
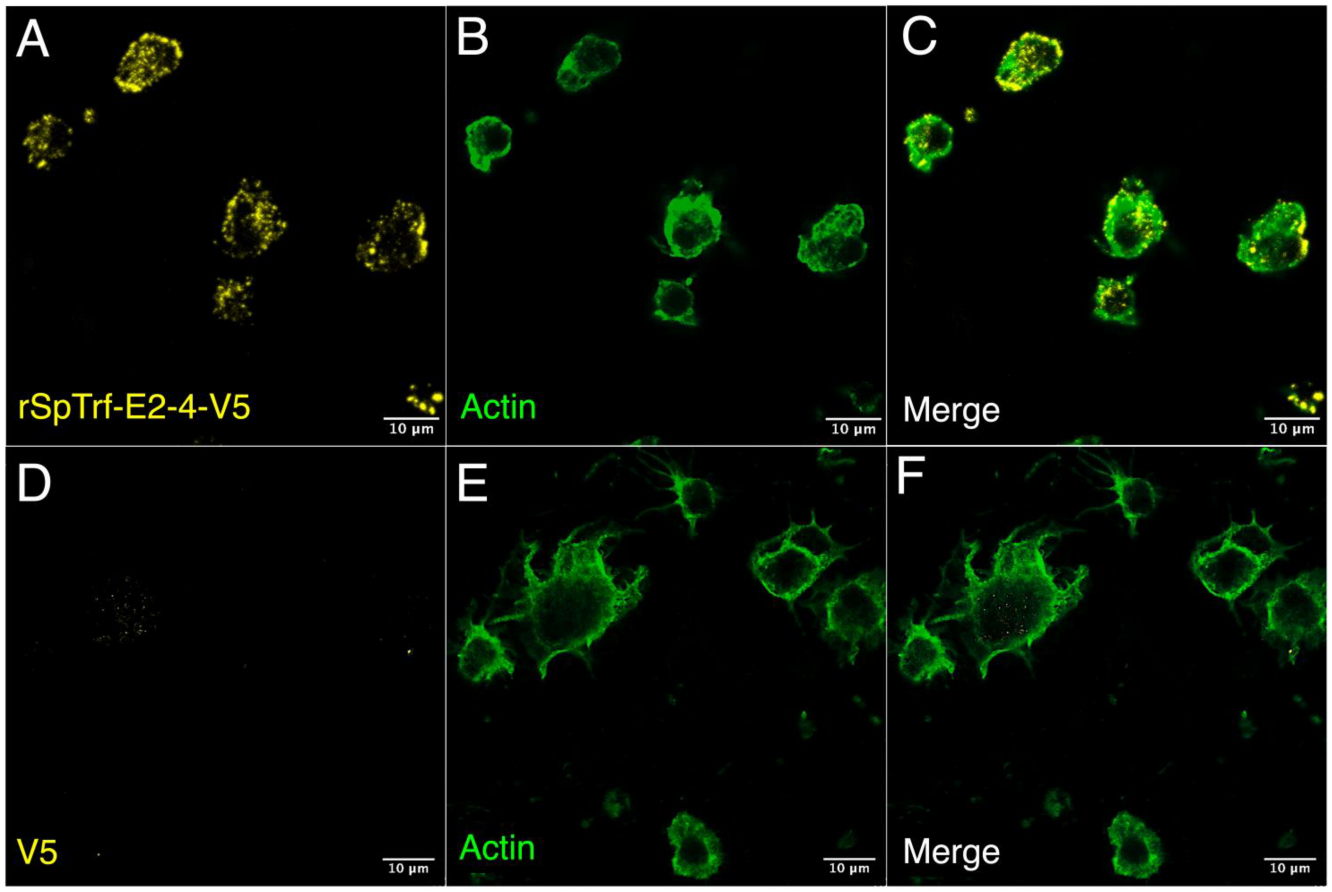
rSpTrf-E2-4 binds to fixed phagocytes. Fixed, impermeable phagocytes were incubated with rSpTrf-E2-4, permeabilized, and labeled with ChαV5 and MαActin followed by GαChIg-405 and GαMIg-488. **(A–C)** Phagocytes bind rSpTrf-E2-4 in a pattern that is distributed over the cell surface with most of the binding near the periphery of the cells. **(D–F)** Fixed phagocytes incubated as in **(A**–**C)** but without rSpTrf-E2-4 do not show background labeling with ChαV5 and GαChIg-405. MαActin and GαMIg-488 label the cytoskeletal structure to identify cell type. Imaging was carried out on an LSM 800 confocal microscope (Zeiss) with false color editing using associated imaging program. Scale bars indicate 10 µm.

Although live phagocytes bound soluble rSpTrf-A6 as well as rSpTrf-A6 cross-linked to inert beads (48), it was not known whether the rSpTrf proteins would bind to fixed cells, particularly given that fixatives often change cell surfaces. Moreover, fixation would negate any active interactions on the surface of live coelomocytes or whether secreted co-factors were required for rSpTrf protein binding. To address this, phagocytes in glass bottom 96-well plates were fixed but not permeabilized and incubated with or without soluble rSpTrf-E2-4 at rt. After incubation, cells were permeabilized and labeled with ChαV5 to visualize the location of rSpTrf-E2-4, and with MαActin to visualize cytoskeletal morphology and identify phagocyte types. Results showed that rSpTrf-E2-4 bound to fixed phagocytes, whereas the control cells were negative for ChαV5, thus confirming the specificity of rSpTrf-E2-4 binding (**Figure 4**). The rSpTrf-E2-4 bound to polygonal phagocytes was primarily distributed over the entire surface of the cell, with a punctate binding pattern at the edges (**Figures 4A–C**; **Supplementary Figures S1C-E**). This binding pattern was consistent, with beads cross-linked to rSpTrf proteins that bound over the surface of a subset of fixed cells (48). Conversely, these binding patterns differed from those observed with live cells, which exhibited rSpTrf-A6 bound in the perinuclear region with no binding at the cell edges (**Figures 2**, **3**; **Supplementary Figures S1A, B**). Overall, the rSpTrf proteins bound to fixed cells, although the binding patterns were different for live compared to fixed cells.

### 3.2 rSpTrf proteins do not bind to red spherule cells

**Figure 5:**
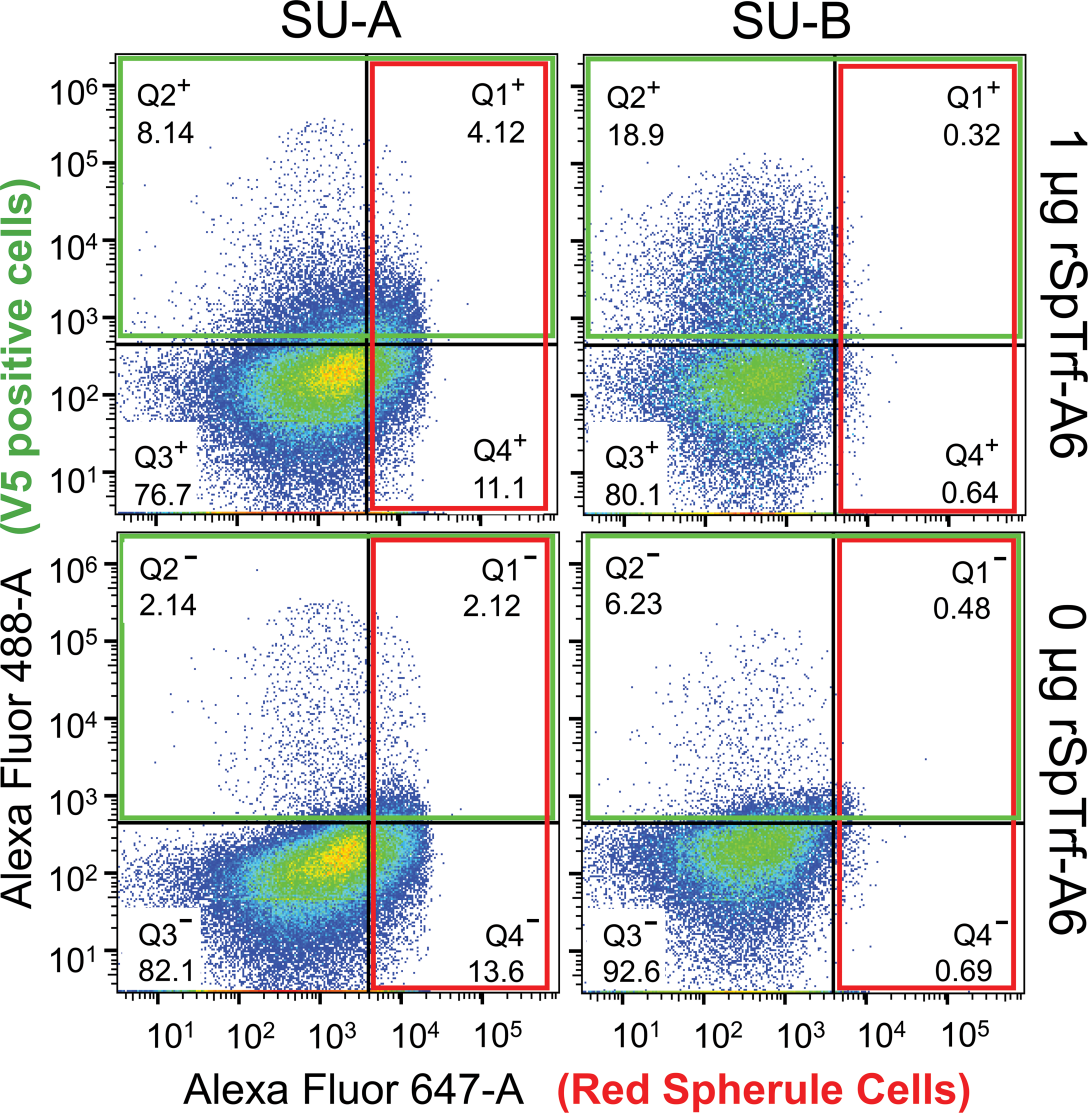
rSpTrf-A6 binds to phagocytes but not red spherule cells. Coelomocytes from two sea urchins (SU-A, SU-B) were incubated with or without rSpTrf-A6 followed by labeling with MαV5-488. Washed cells were evaluated by flow cytometry with a BD Celesta Cell Analyzer based on gates optimized for coelomocytes as reported previously (30, 63, 68). Cells were evaluated for fluorescence with the 488 nm laser to detect MαV5-488 (boxed in green), and the 647 nm laser to detect the autofluorescence of the red spherule cells (boxed in red). Detection of red spherule cells in Quadrats 1 and 4 (Q1 and Q4) in each of the four scatter plots was established based on gating profiles from Yakovenko et al. (30). Q1 and Q2 were defined based on the fluorescence levels of negative control cells incubated without rSpTrf-A6 (two lower scatter plots) as detected by the 488 nm laser. The percentage of cells in each quadrat is indicated. For gating details, see **Supplementary Figure S4**.

When coelomocytes are spun or settled onto glass slides, the phagocytes are the only coelomocytes that attach tightly enough to remain on the slide through subsequent washes and processing (29, 42, 52). Although the results from the cytology studies indicated that the rSpTrf proteins bound to phagocytes (**Figures 2**–**4**; **Supplementary Figure S1**), this did not address the question of whether other types of coelomocytes could bind the rSpTrf proteins. Furthermore, there are conflicting reports as to whether the non-phagocyte classes of coelomocytes express or interact with native Trf proteins (30, 44). To address this, live coelomocytes from two sea urchins (SU-A, SU-B) were incubated in cold aCF with or without rSpTrf-A6, labeled with MαV5-488, and analyzed by flow cytometry using gates established for coelomocytes from both *S. purpuratus* (**Supplementary Figure S4**) (63) and *Paracentrotus lividus* (30). This approach discerned whether red spherule cells, which are distinguished based on their red autofluorescence (**Figure 5**, quadrat (Q)1^+^ and Q4^+^), also bound rSpTrf-A6 as well as permitting a comparison to rSpTrf-A6-binding by other types of coelomocytes (**Figure 5**, Q1^+^ and Q2^+^). As previously reported, colorless spherule cells and vibratile cells cannot be distinguished by this approach (68). Although coelomocytes from each sea urchin displayed similar overall subset proportions, there were slight differences in the proportion of cells that bound rSpTrf-A6. SU-A had fewer coelomocytes that bound rSpTrf-A6 compared to SU-B (8.14% vs 18.9%, respectively) (**Figure 5**, Q2^+^), and there were differences in anti-V5 background labeling for SU-A and SU-B (2.14% and 6.23%, respectively) (**Figure 5**, Q2**^−^**). For both animals, the greatest number of rSpTrf-A6 positive cells were identified in Q2^+^ (**Figure 5**) and when the background for anti-V5 was subtracted (cells labeled with anti-V5 in the absence of rSpTrf-A6), negligible labeling was observed for red spherule cells that were incubated with rSpTrf-A6 (**Figure 5**, Q1^+^). Notably, co-incubation of coelomocytes from either animal with rSpTrf-A6 resulted in significant changes to the scatter profiles (Chi-square test; *p* < 0.05) for cells incubated with rSpTrf-A6 (Q1^+^ - Q4^+^) compared to those without (Q1**^−^** - Q4**^−^**). It was also noteworthy that SU-B, which had more cells that bound rSpTrf-A6, had a relatively low number of red spherule cells, corroborating that red spherule cells did not bind rSpTrf proteins and previous observations that polygonal and small phagocyte populations bound (and phagocytosed) beads cross-linked with rSpTrf proteins (48).

### 3.3 rSpTrf binding to phagocytes is variable

**Figure 6:**
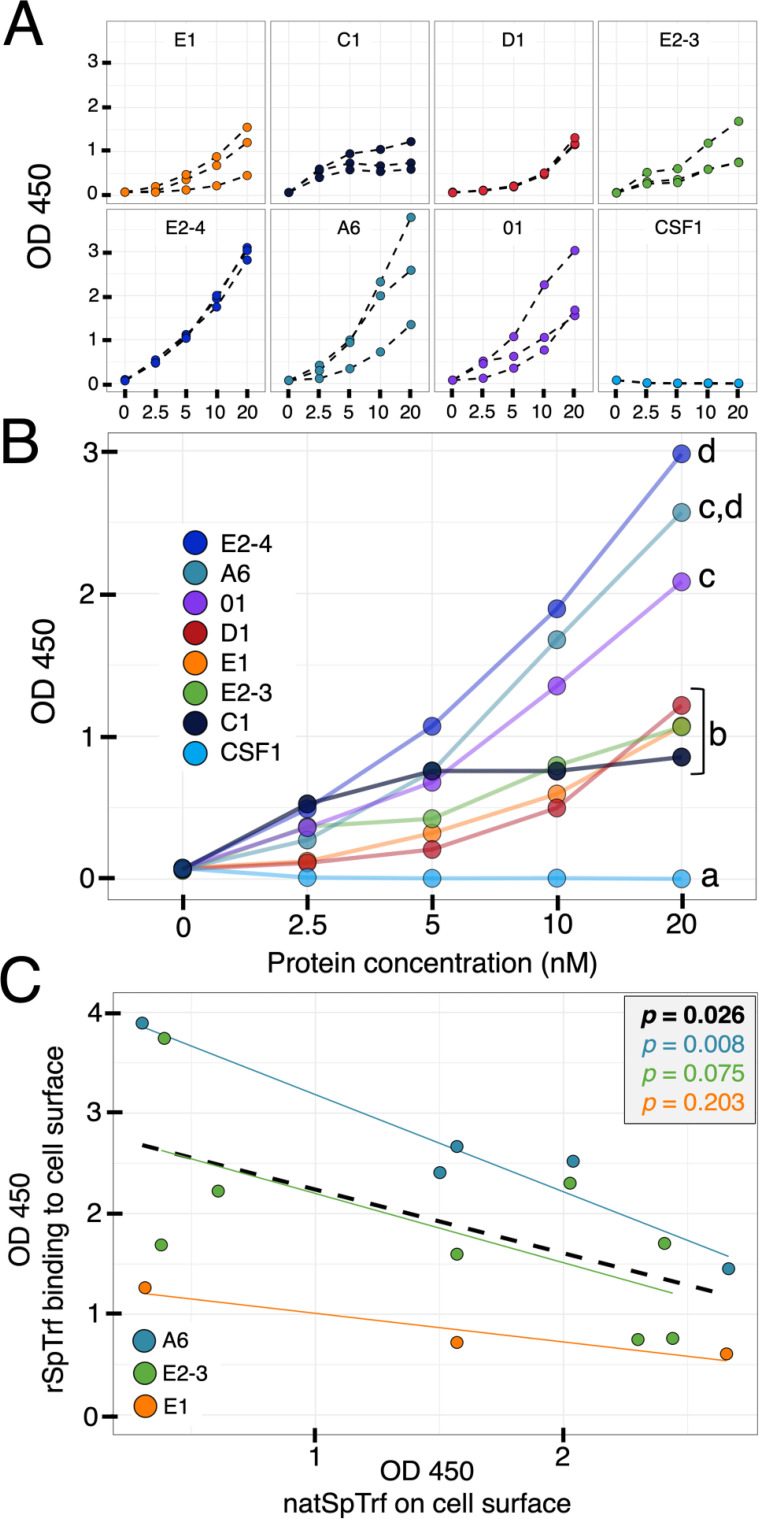
The rSpTrf proteins show distinct levels of binding to phagocytes. The rSpTrf proteins and rCSF-1 (negative control) were incubated with cells collected from three different sea urchins. Binding was evaluated with MαV5-HRP using In-cell ELISA. **(A)** The rSpTrf proteins bind to phagocytes. Each rSpTrf protein shows increased binding with increasing concentration except for rSpTrf-C1, which shows a binding plateau. Cells from the three sea urchins show binding variability that is also dependent on the rSpTrf protein. No binding is evident for rCSF-1. **(B)** There are significant differences in cell binding among the rSpTrf proteins. The binding curves in panel **(A)** were fitted with a linear regression model (*y = mx + b*) to estimate the marginal effect of protein concentration on the change in OD^450^. The marginal effects for each protein were compared to each other, and statistically significant differences were established using a Tukey test with significance set at *p* < 0.05. Letters indicate significant differences, and shared letters indicate no differences. rSpTrf-E2-4, -A6, and -01 show the steepest slopes and thus exhibit significantly more binding. In contrast rSpTrf D1, -E2-3, and -C1 have shallower slopes, indicating significantly less binding. Binding by all rSpTrf proteins is higher than rCSF-1, which does not bind to the cells. **(C)** Elevated levels of natSpTrf proteins on cell surfaces correlate negatively with rSpTrf binding to cells. Coelomocytes in separate wells were evaluated for natSpTrf proteins with α-71 and GαRIg-HRP and for rSpTrf proteins with MαV5-HRP in separate wells using In-cell ELISA. The level of natSpTrf proteins on the surface of coelomocytes is negatively correlated with binding by the rSpTrf proteins to the same coelomocytes (black dashed line; *p* ≤ 0.05; Pearson’s correlation test). Differences exist among the rSpTrf proteins that were tested. The high level of binding by rSpTrf-A6 **(A, B)**, has an inverse relationship between the level of natSpTrf proteins on the surface of coelomocytes tested for rSpTrf-A6 binding. rSpTrf-E1 has a low level of binding **(A, B)** and has an inverse relationship with natSpTrf on cells. The result for rSpTrf-E2-3 is intermediate.

The qualitative analysis of rSpTrf-A6 and -E2-4 verified that these proteins bound to the surface of phagocytes (**Figures 2**–**4**; **Supplementary Figure S1**), however these results did not quantify the extent of binding or the binding potential of the other rSpTrf proteins. Although distinct rSpTrf proteins cross-linked to beads show different capacities to enhance phagocytosis (48), it is not known whether these variations arise from differences among phagocytes for binding rSpTrf proteins (48), differences in the binding capacities of rSpTrf proteins that may be dictated by their sequence variations, or both. Because both rSpTrf-A6 and -E2-4 bound to cells, quantification of binding by all of the proteins was addressed by In-cell ELISA. Preliminary evaluation of coelomocytes by In-cell ELISA indicated that rSpTrf-E2-3 bound both fixed and live coelomocytes (**Supplementary Figure S5**), consequently fixed cells were used for subsequent assays. Cells collected from three sea urchins were spun onto wells of a 96-well plate, fixed but not permeabilized, and incubated with increasing concentrations of each rSpTrf protein. rCSF-1, which was produced by same method as the rSpTrf proteins and bearing a V5 tag (69), was used as an irrelevant protein control. Background levels were determined from wells to which no cells were bound. The level of protein binding in wells was measured using MαV5-HRP followed by measuring the absorbance at OD^450^. All proteins exhibited increased cell binding with increasing protein concentration compared to the negative control, rCSF-1, which did not show detectible binding to coelomocytes at any concentration (**Figure 6A**). Furthermore, the rSpTrf proteins did not bind to fixed Sf9 insect cells (**Supplementary Figure S6**), confirming their specificity for binding to sea urchin coelomocytes. Comparisons among the rSpTrf proteins indicated distinct levels of binding in addition to variability in binding to cells from different sea urchins. rSpTrf-A6 and -01 showed relatively greater variation in binding to cells from the three animals, which was most evident at 20 nM (**Figure 6A**). Alternatively, rSpTrf-E2-4 and -D1 showed almost comparable levels of binding to cells collected from each of the animals. There were also differences in the apparent binding saturation among the proteins, as inferred by a binding plateau observed for some of the rSpTrf proteins. Binding by rSpTrf-C1 plateaued at 5 nM with little increase at higher concentrations of protein. Binding by rSpTrf-E2-3 to cells from two animals appeared to reach saturation at 10 nM, but did not plateau for cells from the other sea urchin. Binding by the remaining proteins (rSpTrf-E1, -D1, -E2-4, -A6, and -01) did not plateau for the concentration range that was employed indicating that saturation had not been reached. The results for rSpTrf binding to cells suggested that variations may be due to sequence differences among the proteins in addition to influences from cells collected from different sea urchins.

Significant differences in cell binding among the different rSpTrf proteins was determined using linear regression. A linear model was used to estimate the marginal effect of protein concentration (nM) on the change in OD^450^. This marginal effect was compared among rSpTrf proteins to determine whether the effect of protein concentration on change in absorbance at OD^450^ was significantly different among the rSpTrf proteins. Results indicated that the variations in binding by the rSpTrf proteins could be categorized into two levels based on comparisons among the slopes of binding associated with increasing concentrations of rSpTrf proteins (**Figure 6B**). rSpTrf-E2-4, -A6, and -01 showed the highest capacities for binding, with significantly (Tukey test, *p* < 0.05) steeper slopes compared to results for rSpTrf-E1, -C1, -D1, and -E2-3 that showed lower binding capacity. The on-off binding for rSpTrf-A6 and -E2-3 were evaluated by incubating biotinylated (B-)rSpTrf-A6 or B-rSpTrf-E2-3 with phagocytes followed by incubation with increasing concentrations of the same unlabeled protein. The level of B-rSpTrf protein bound to the cells was measured to determine whether increasing concentrations of the unlabeled rSpTrf protein resulted in a displacement and decrease in the bound biotinylated version. Results showed no change in the level of bound B-rSpTrf-A6 or B-rSpTrf-E2-3 when competed with unlabeled versions (**Supplementary Figure S7**). Overall, these results suggested that all rSpTrf proteins bound tightly to the surface of phagocytes with very low on-off rates, but with variations in both the level of binding and the concentration at which binding saturation occurred.

### 3.4 rSpTrf binding to cells is highest when cell surface natSpTrf proteins are low

The polygonal and small phagocytes are the major coelomocytes that produce the natSpTrf proteins, which are localized in small perinuclear vesicles, and on the surface of the small phagocytes (42, 44, 52). The number of small phagocytes and their natSpTrf protein expression in the CF increases with immune activation in response to LPS or *Vibrio diazotrophicus* (42, 44). Therefore, the level of natSpTrf proteins on the surface of small phagocytes can be used as a proxy for the immune status of individual sea urchins. Furthermore, the majority of secreted natTrf proteins in several sea urchin species tend to multimerize upon collection (30, 42, 43, 70), suggesting that natTrf proteins interact with each another in homotypic and heterotypic multimers. Because coelomocytes from different sea urchins were used for the In-cell ELISA assays, there was likely variation in both the level of natSpTrf proteins on the small phagocytes and the immune status of the animals. Consequently, we investigated the relationship between the level of cell surface natSpTrf proteins on phagocytes from individual animals, which were also employed in parallel to detect binding by rSpTrf-A6, -E2-3, and -E1. Results were evaluated by measuring the level of cell surface binding of rSpTrf proteins with anti-V5 compared to the level of natSpTrf proteins observed on the surface of the small phagocytes (in separate wells) using the average readout from α-71, an anti-natSpTrf antibody. Because cells were fixed immediately after collection but not permeabilized before exposure to experimental conditions, the level of cell surface natSpTrf on the small phagocytes from each animal could be quantified and correlated with the level of binding soluble rSpTrf proteins. This approach differed from comparisons by cytology in which natSpTrf levels were quantified after live cells were collected and incubated with rSpTrf proteins (**Supplementary Figure S2**). Results showed a significant negative correlation (*p* = 0.026, Pearson’s correlation test) between the level of rSpTrf cell surface binding relative to the level of natSpTrf proteins on cells from the same animal (**Figure 6C**). However, the correlation between the level of natSpTrf proteins and binding by the different rSpTrf proteins was variable, with rSpTrf-A6 showing the most negative correlation, followed by rSpTrf-E2-3, and rSpTrf-E1, which was consistent with variable cell binding levels for these three proteins (**Figure 6B**). Results indicated that the level of rSpTrf protein bound to cells was high when the natSpTrf protein on the same cells was low.

### 3.5 The rSpTrf proteins bind phagocytes with specificity and compete for cognate binding site(s)

**Figure 7:**
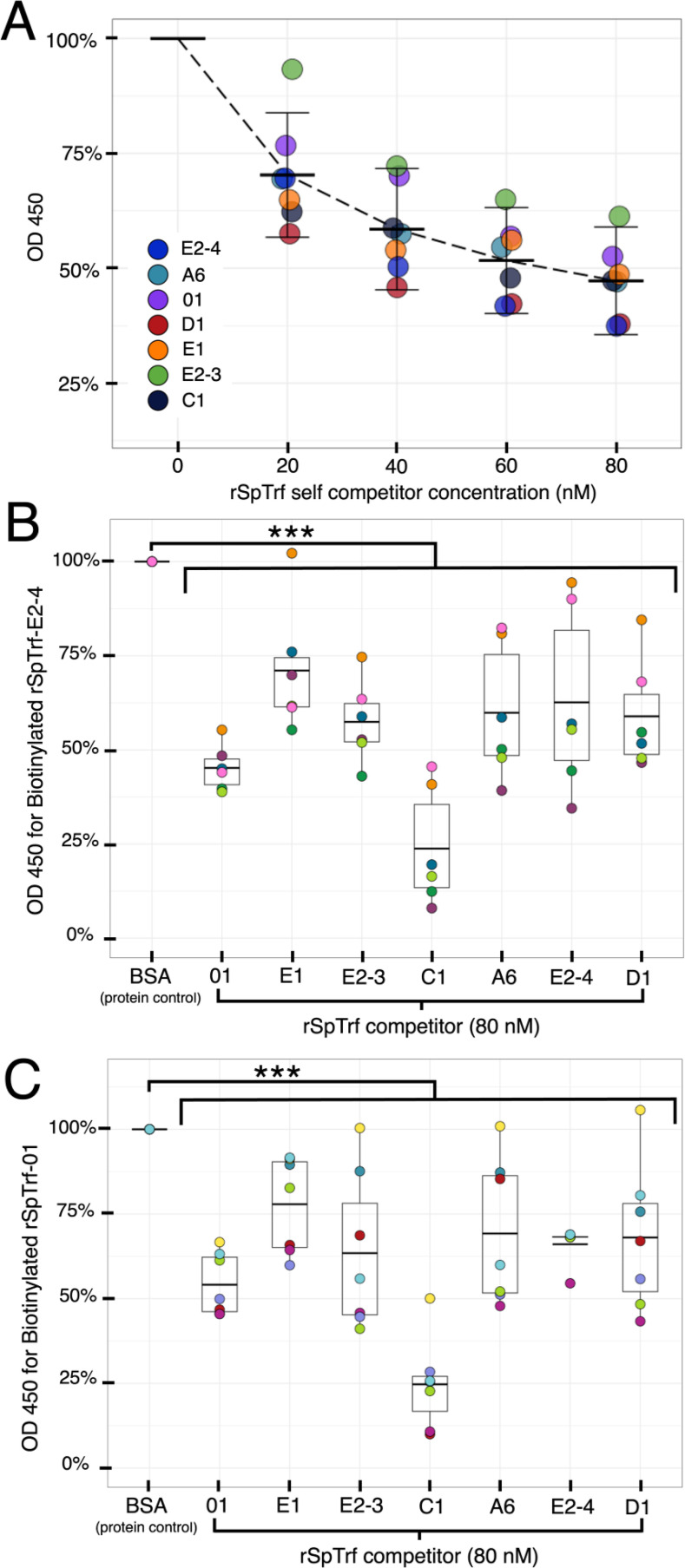
The rSpTrf proteins show specific binding to phagocytes. Binding specificity by rSpTrf proteins to fixed cells in wells is evaluated by self-competition and cross-competition with other rSpTrf proteins. **(A)** Each rSpTrf protein competes with itself for binding to phagocytes. B-SpTrf proteins (20 nM) mixed with increasing concentrations of the same but unlabeled rSpTrf protein were incubated with coelomocytes. Cell binding by the biotinylated proteins was quantified with streptavidin-HRP using In-cell ELISA. Results were normalized to protein binding in the absence of a competitor, which was set to 100%. Each protein shows decreased binding in self-competition assays. **(B)** Cell binding by B-SpTrf-E2-4 is competed by each of the unlabeled rSpTrf proteins. B-rSpTrfE2-4 (20 nM) mixed with each of the rSpTrf proteins (80 nM) or BSA (80 nM) was evaluated for cell binding with streptavidin-HRP using In-cell ELISA. Cross competition binding results were normalized to the competition with the irrelevant control protein, BSA, which was set to 100%. All rSpTrf proteins compete and reduce significantly binding of B-rSpTrf-E2-4 to cell surfaces. **(C)** Cell binding by B-SpTrf-01 is competed by each of the unlabeled rSpTrf proteins. Cross competition was repeated as described for panel **(B)** using B-rSpTrf-01. Results were similar; all rSpTrf proteins compete with B-rSpTrf-01 for binding to cells. Significant reductions (*p* < 0.01) in binding shown in **(B, C)** were determined using Dunnett’s test. ***, *p* < 0.001.

The rSpTrf proteins showed variable levels of binding to the surface of phagocytes, which suggested that the different isoforms either have disparate binding sites on sea urchin cells, or they have different capacities for binding to the same site(s) (**Figures 6A, B**). To address this question, and to confirm that binding was specific, each of the B-rSpTrf proteins was evaluated for binding specificity based on competition with the same respective protein using In-cell ELISA. To determine whether there was a specific binding site for each rSpTrf protein, fixed but non-permeabilized phagocytes from three different sea urchins were incubated with a mixture of each B-rSpTrf protein and increasing concentrations of the same respective unlabeled rSpTrf protein. The level of binding by the B-rSpTrf proteins were evaluated with streptavidin-HRP and normalized to the level of binding in the absence of a competitor that was set to 100%. Results indicated a relatively similar reduction in B-rSpTrf protein that bound to cells correlated with increasing concentrations of self-competitor, with an average maximum reduction to 47.5% (± 11.7%) (**Figure 7A**). This suggested that all of the rSpTrf proteins competed with themselves for binding and that each protein bound with specificity to the cell surface.

To determine whether there was a shared or cognate binding site for the rSpTrf proteins, B-rSpTrf-E2-4 and B-rSpTrf-01 were chosen for evaluation in cross-competition assays against the other rSpTrf proteins that included comparisons to self-competition. The level of B-rSpTrf protein binding was normalized against the binding level when in competition with BSA, which served as the negative control. Self-competition with rSpTrf-E2-4 and -01 reduced binding of B-rSpTrf-E2-3 and B-rSpTrf-01 (**Figures 7B, C**) in agreement with increasing concentration of the competitor and binding specificity by the rSpTrf proteins (**Figure 7A**). Furthermore, cross-competition between the B-rSpTrf proteins and each of the other rSpTrf proteins resulted in significant reductions (*p* < 0.01, Dunnett’s test) in binding for the B-rSpTrf proteins compared to competition with BSA (**Figures 7B, C**). The diminished binding observed in these competition assays suggested that all of the rSpTrf proteins were binding specifically to the same site(s) on phagocytes. These results also corresponded with reduced binding by rSpTrf proteins on cells with higher levels of cell surface natSpTrf proteins (**Figure 6C**), suggesting that both the rSpTrf and natSpTrf proteins competed for the same binding site(s) on phagocytes.

### 3.6 rSpTrf binding to phagocytes modulates immune gene expression

**Figure 8:**
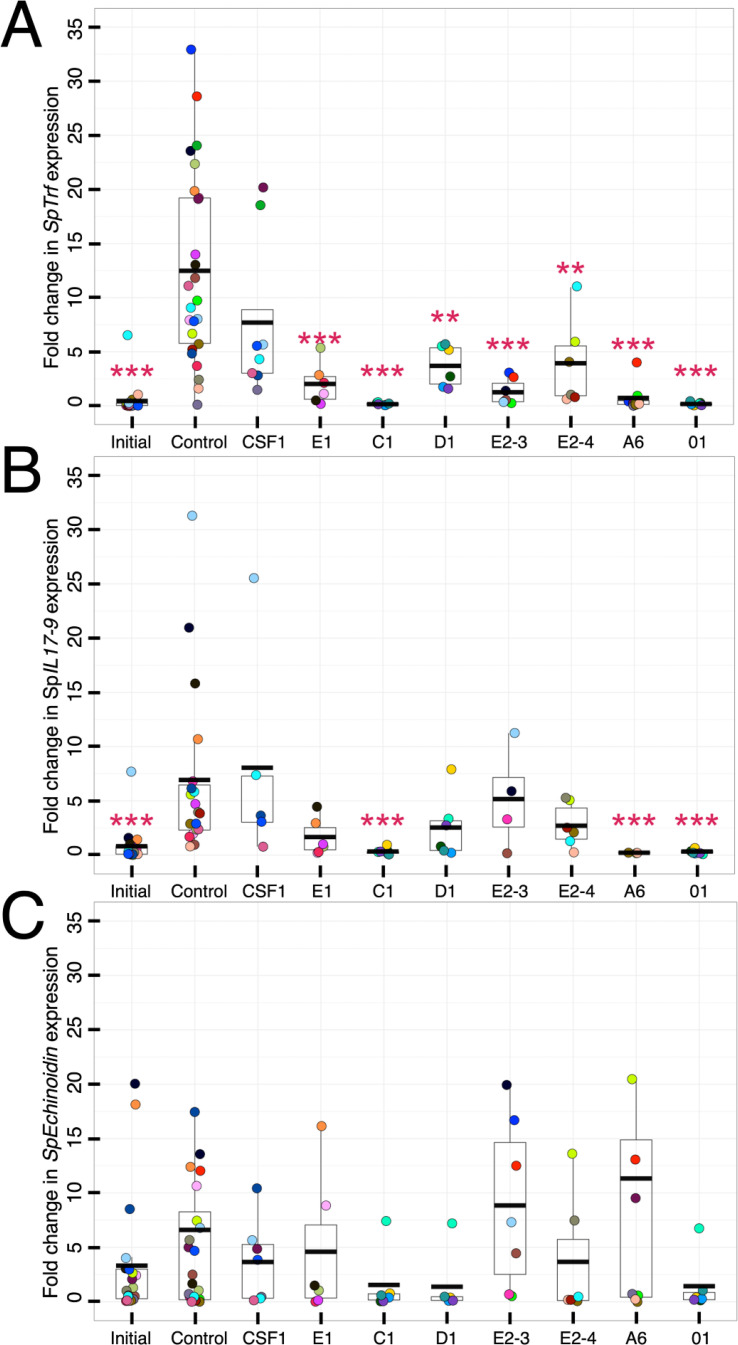
Phagocytes with bound rSpTrf proteins modulate expression of the *SpTrf* gene family and *SpIL17-9*. Soluble rSpTrf proteins incubated with coelomocytes from at least six sea urchins in short term cultures maintain low expression of the *SpTrf* genes and *SpIL17-9*, but not *SpEchinoidin*. Coelomocytes were either processed immediately (initial) for total RNA isolation, or were incubated with each of the rSpTrf proteins, with rCSF-1 (protein control), or without added proteins (negative control). Total RNA was isolated after 4 hours of incubation, processed, and evaluated for expression of selected genes by qRT-PCR. Results were normalized to the housekeeping gene *SpL8* which encodes the large ribosomal protein 8 (71). Fold changes in gene expression were compared to the negative control, without added protein, and evaluated for significant differences (*p* ≤ 0.01, Dunnet’s test). **(A)** Each rSpTrf protein maintains low expression of the *SpTrf* gene family. Cells incubated with the rSpTrf proteins maintain low expression of the *SpTrf* gene family that is similar to the initial level of expression of the cells. In the absence of added protein or with rCSF-1, *SpTrf* expression is elevated. **(B)** A subset of rSpTrf proteins maintain low expression of *SpIL17-9*. Cells incubated with rSpTrf-01, -C1, -A6, and -01 maintain low expression of the *SpIL17-9* that is similar to the initial level of expression. Expression after incubation with the other rSpTrf proteins is more random among cells from different sea urchins and is not different from the negative control or incubation with rCSF-1. **(C)** The rSpTrf proteins do not impact expression of *SpEchinoidin*. Cells incubated with the rSpTrf proteins do not show significant changes in the expression of *SpEchinoidin* compared to the initial expression level, to cells incubated with rCSF-1, or to the negative control cells incubated without protein. The colors of each dot in each of the panels indicates results for cells collected from individual sea urchins. **, *p* < 0.01; ***, *p* < 0.001.

Immune stimulation of sea urchins results in substantial increases in *SpTrf* gene family expression, lending strong inference that the encoded proteins function in echinoid immune responses [(32, 33, 38); reviewed in (31)]. rSpTrf-E1-Ec binds to an array of foreign targets and PAMPs (41, 61) and the natSpTrf proteins function as opsonins and augment coelomocyte phagocytosis of a marine *Vibrio* (46). Moreover, soluble rSpTrf proteins bind directly to small and polygonal phagocytes (**Figures 2**–**4**; **Supplementary Figure S1**) and facilitate phagocytosis of cross-linked beads (48). However, the cellular consequences of rSpTrf binding to phagocytes have not been addressed. Accordingly, the responses of rSpTrf-bound phagocytes were assessed through gene expression analysis. Coelomocytes were collected from individual sea urchins and total RNA was isolated immediately after collection from a subset of the cells to assess initial levels of gene expression. The remainder of the cells were incubated with each of the respective rSpTrf proteins, with rCSF-1, or without added protein. After 4 hours, the relative expression of key immune response genes were examined by qPCR relative to the housekeeping control gene, *SpL8*, which encodes protein 8 that functions in the large subunit of ribosomes (52, 71). The immune genes included members of the *SpTrf* gene family to determine whether rSpTrf proteins bound to cell surfaces altered their own expression. *SpIL17-9* was chosen because it encodes an orthologue of the vertebrate pro-inflammatory interleukin cytokine IL17, that is upregulated in coelomocytes in response to immune challenge (62). *SpEchinoidin* encodes a C-type lectin and was also chosen because it is expressed in response to immune challenge (71) and has been employed previously to detect immune activation in sea urchin coelomocytes (38). Gene expression by cells incubated with each of the different rSpTrf proteins, the irrelevant protein control rCSF-1, and cells that were processed upon collection to determine the initial level of gene expression, were compared to cells incubated without added proteins. Cells incubated without added protein or with rCSF-1 showed significantly elevated expression for the *SpTrf* gene family and *SpIL17-9* compared to the level of initial expression (**Figures 8A, B**). This aligns with previous findings that sham injections or coelomocyte collection trigger injury responses (32, 38). Similarly, cells incubated with rCSF-1 exhibited elevated expression of the *SpTrf* gene family and *SpIL17-9*, which was not significantly different from expression in cells incubated without added protein. Surprisingly, incubation with the rSpTrf proteins resulted in significantly lower (*p* < 0.01, Dunnet’s test) expression of the *SpTrf* gene family and *SpIL17-9* compared to the control treatment (no added protein) and to cells incubated with rCSF-1 (**Figures 8A, B**). Furthermore, expression was similar to that in freshly isolated coelomocytes. Coelomocytes incubated with rSpTrf-C1, -A6, or -01 showed significantly lower expression of *SpIL17-9* compared to control cells exposed to rCSF-1 or to cells without added protein (**Figure 8B**). Conversely, coelomocytes incubated with rSpTrf-E1, -D1, -E2-3, or -E2-4 did not show significant differences in *SpIL17-9* expression relative to the control treatments, albeit with greater expression variability observed among treatments (**Figure 8B**). *SpEchinoidin* expression was highly variable and not significantly different among any of the treatments (**Figure 8C**). It was notable, however, that cells from all but one sea urchin had low *SpEchinoidin* expression following treatment with rSpTrf-C1, -D1, and -01, compared to non-treated control cells (**Figure 8C**). Together, these findings suggested that the soluble rSpTrf proteins influenced the expression of their own gene family and may have immuno-modulatory activity in addition to their noted functions of immune responsiveness and opsonization.

## 4 Discussion

The natSpTrf proteins in sea urchins clearly have diverse functions in the innate immune system of these animals. rSpTrf proteins direct the phagocytosis of opsonized targets, as demonstrated by the enhanced binding and phagocytosis of inert beads cross-linked to rSpTrf proteins (48). Use of inert beads avoids the confounding effects of PRR mediated phagocytosis when microbes are used as foreign targets. The several rSpTrf proteins evaluated here show variability in binding to phagocytes and their impact on the expression of the *SpTrf* gene family and the *SpIL17-9* gene. Differences in their activities are not correlated with their sequences and element patterns including the two isolates of the rSpTrf-E2 protein. It is notable that the result for rSpTrf-E1 that shows very low levels of phagocytosis when cross-linked to inert beads (48), is in agreement with rSpTrf-E1-Ec that binds to *V. diazotrophicus* but does not enhance phagocytosis (46). These results suggest that the natSpTrf proteins have a range of functions *in vivo*, including activities other than opsonization. Because rSpTrf-E1-Ec transforms from disordered to α helical upon binding LPS and other targets (47), this suggests that structural transformations occur when natSpTrf proteins bind foreign targets, presumably enabling recognition and binding by the phagocytes. Yet, it is not known whether the rSpTrf proteins produced by the eukaryotic expression system (Sf9 insect cells) undergo conformational changes when cross-linked to beads. If the rSpTrf proteins remain disordered when cross-linked to bead surfaces, phagocytes may recognize these proteins without any conformational change, potentially resulting in distinct cellular responses depending on whether they detect disordered or transformed natSpTrf proteins. The soluble rSpTrf proteins show variable binding capabilities yet they bind specifically and tightly to the surfaces of the small and polygonal phagocytes, which is consistent with putative shared binding site(s) for the rSpTrf proteins. Furthermore, once bound by phagocytes in the absence of foreign targets or PAMPs, the complex of rSpTrf proteins and binding site(s) appear to aggregate and/or be endocytosed. Isolation of coelomocytes from sea urchins induces immune activation in these cells whereas binding rSpTrf proteins dampens these responses, including the *SpTrf* gene family, as well as at least one other immune-related gene. Activities of the rSpTrf proteins infer broad functions of the natSpTrf proteins *in vivo* that includes opsonization, structural transformation, phagocytosis, and modulation of the innate immune response.

### 4.1 Soluble rSpTrf proteins bind to subtypes of phagocytes

The phagocytes have been proposed as the primary coelomocytes that control cellular immune functions *in vivo* (51). This is consistent with the small and polygonal phagocytes that interact with rSpTrf proteins cross-linked to beads (48) and with natSpTrf proteins bound to foreign targets (46). rSpTrf proteins do not bind to other classes of coelomocytes, as shown here (**Figure 5**) and reported previously (44). The localization of bound rSpTrf proteins is different depending on whether the phagocytes are fixed or live, and show a distributed pattern or an aggregated, perinuclear pattern, respectively. This difference is consistent with movement of the rSpTrf proteins and the binding site(s) on live cells from general distribution including the edges of the cells to an aggregated and/or endocytosed distribution in the perinuclear region. Receptor aggregation is a common feature in immune system signaling such as Toll-like receptors, which cluster after binding ligands to form large complexes that initiate cell signaling (72). The aggregation of the rSpTrf proteins and possible endocytosis, protein sorting by the endosomal compartment, and return of the binding site(s) to the cell surface are consistent with the activation of a signaling pathway that may regulate gene expression (73).

### 4.2 The rSpTrf proteins have distinct binding capacities

The diversity of the SpTrf proteins has led to the hypothesis that isoforms with different element patterns and sequences exhibit functional differences [(41, 46) reviewed in (31)]. Indeed, some of the rSpTrf proteins (rSpTrf-E2-3, -E2-4, -A6, and -01) enhance phagocytosis of beads, whereas others (rSpTrf-C1 and -D1) have intermediate capabilities for eliciting phagocytosis (48). Variations in cell binding by soluble rSpTrf proteins generally aligns with their opsonin functions when cross-linked to beads, with highest cell binding and enhanced phagocytosis for rSpTrf-E2-4, -A6, and -01, and significantly lower binding and phagocytosis for rSpTrf-E1, -E2-3, -C1, and -D1 (**Figure 6**). Based on these comparisons and by employing transformed *p*-values to rank the rSpTrf proteins for cell binding, their element patterns do not correlate with their level of binding to phagocytes (**Supplementary Text S1**; **Supplementary Figures S8**, **S9**; **Supplementary Tables S2**, **S3**). It is noteworthy that the two isolates of rSpTrf-E2 with identical sequences display differences in cell surface binding, but show similarly enhanced phagocytosis when cross-linked to beads (48). This is in contrast to rSpTrf-E1 that shows consistent activity irrespective of whether the recombinant protein is expressed by prokaryotes or eukaryotes (46, 48). In general, variations in the functions of the rSpTrf proteins reported here and previously (41, 48) are not correlated with protein element pattern and sequence.

The rSpTrf proteins exhibit different apparent binding saturation levels in which rSpTrf-C1 reaches a binding plateau at low concentrations whereas the other proteins do not share this characteristic at similar concentrations. Although it would be informative to establish binding saturation curves to calculate binding constants for individual rSpTrf proteins or to estimate the numbers of receptors on individual cells, there are several reasons why this is not possible. Sea urchins that provide the coelomocytes are collected from a large, outbred, genetically diverse population (74), which likely impacts the functions of the cells and how they may respond to the rSpTrf proteins. Differences in the immune status of individual sea urchins is commonly reflected in variations in the proportions of coelomocyte subpopulations [(30, 51, 52); reviewed in (50)]. The putative binding site(s) for the SpTrf proteins are unknown and may be expressed at variable levels among different types of phagocytes based on differences in genetics, differences in the population sizes of the phagocytes, and the immunological status of the animal. Collectively, these aspects may impact variations in individual rSpTrf protein binding to cells from different sea urchins. Other unaccounted variables may also contribute to the inability to estimate the number of binding sites and to keep binding sites constant for determining binding constants. To overcome some of these limitations the same number of cells from different individual animals were evaluated for binding. One notable attribute of this system that appears to be constant is the very low on-off rate for rSpTrf-E2-3 and -A6 bound to the cells. This characteristic likely applies to the other rSpTrf proteins and to the natSpTrf proteins *in vivo*; once bound to the cell surface, they do not dissociate quickly.

Binding by the rSpTrf proteins is impacted by the level of cell surface natSpTrf proteins that are displayed on the phagocytes. This, in turn, can vary depending on the proportion of small phagocytes relative to polygonal phagocytes in the cell populations from individual sea urchins. A previous hypothesis stated that natSpTrf proteins on the surface of small phagocytes may multimerize with natSpTrf proteins opsonized to microbes or other foreign targets to promote phagocytosis (42). This notion is consistent with a reduction in phagocytosis of bacteria by coelomocytes from *Paracentrotus lividus* when the coelomocytes are pre-incubated with anti-natSpTrf antibodies (30). This infers that native Trf proteins in *P. lividus* (natPlTrf) on the surface of phagocytes are blocked by the antibodies from interacting directly with the natPlTrf proteins opsonized on the bacteria. However, the negative correlation between the level of cell surface natSpTrf proteins and the level of soluble rSpTrf-A6 bound to phagocytes presented here is not consistent with this interpretation. Our findings suggest that soluble rSpTrf proteins may not interact with small phagocytes that have elevated levels of surface-bound natSpTrf proteins. An alternative interpretation of the anti-natSpTrf antibody used to block binding by the surface natSpTrf proteins on small phagocytes, is that it actually blocks direct interactions with PAMPs on foreign targets. Furthermore, using anti-natSpTrf antibodies to block function does not apply to the polygonal phagocytes because they do not mount natSpTrf proteins on the surface (42, 44). The polygonal phagocytes bind rSpTrf proteins directly, irrespective of whether the proteins are bound to a target (48) or are in soluble form as shown here. Consequently, we propose that natSpTrf proteins on small phagocytes may bind directly to microbes, or these cells and the polygonal phagocytes may bind natSpTrf proteins opsonized to a target by their putative SpTrf binding site(s). Both approaches for binding natSpTrf proteins would lead to phagocytosis by both phagocyte types. This hypothesis illustrates the dual arms of the natSpTrf system, which can function through both humoral and cell-mediated pathways to clear pathogens.

### 4.3 rSpTrf proteins bound to cells leads to immune gene regulation

Characteristics among the rSpTrf proteins include variability in binding to putative cell surface site(s), which may result in distinct cellular responses that are dependent on the conformation of the protein. Previous work identified that rSpTrf-E1-Ec is intrinsically disordered but adopts a more ordered conformation upon binding targets (47, 61). The conformational state of the rSpTrf-E1 proteins bound to cells as transformed opsonins on targets may initiate a pro-inflammatory responses, whereas soluble and disordered proteins bound to cells may initiate anti-inflammatory responses. Distinct cellular immune signaling resulting from variations in ligand conformation are not unique to the rSpTrf proteins in sea urchins. In adaptive immunity, distinct activation or inhibitory signaling arises through the high-affinity Fc receptors that engage either monomeric antibodies or those cross-linked and bound to antigen (75). For example, the high-affinity IgE receptor, FcϵRI, on mast cells binds monomeric IgE, but immune activation and secretion of pro-inflammatory mediators only occurs when the FcϵRI-IgE complex includes a bound antigen (76). Similarly, an anti-inflammatory signal is initiated when FcαRI binds monomeric IgA (77). In both cases, the activation responses depend on the detection of foreign targets, and in their absence responses prevent excessive inflammation and maintain homeostasis. The hypothesis proposed here suggests that the structural conformation of the natSpTrf proteins align with this concept and may have precise regulatory control over immune activation or modulation that is of essential benefit either for host protection or from excessive or errant inflammatory responses.

The findings reported here for rSpTrf protein functions support the concept of multifunctional immune effector proteins that also regulate immune responses relative to the presence or absence of a foreign threat, potentially based on the structural status of the protein upon binding the phagocyte. Phagocytes incubated with soluble rSpTrf proteins maintain low levels of expression by the *SpTrf* gene family and the pro-inflammatory *SpIL17-9* gene. This is very different from coelomocytes incubated in the absence of an rSpTrf protein or with an irrelevant protein that may result in the absence of putative regulatory signaling and the perception of and response to injury (32, 38), with the outcome of up-regulating the *SpTrf* gene family and *SpIL17-9*. This suggests that phagocytes *in vivo* sense and control the levels of natSpTrf proteins in the external milieu and increase *SpTrf* gene expression and protein secretion upon detection of foreign contact, perhaps through PRR signaling, when the extracellular levels of the natSpTrf proteins are low. Conversely, *SpTrf* gene family expression may be down-regulated when the extracellular level of unbound natSpTrf proteins are high and the foreign targets have been cleared. This results in binding to the putative binding site(s) that aggregate, may be endocytosed, and initiate signaling. The apparent autocrine regulation may serve to adjust the immune activation status of coelomocytes by also modulating the expression of *SpIL17-9* that encodes a pro-inflammatory cytokine. In general, these results suggest an unknown immuno-sensing regulatory mechanism in echinoids.

The ability to modulate expression of the *SpTrf* gene family correlates with the cell binding capacity among the rSpTrf proteins. For example, rSpTrf-C1 shows binding saturation at low concentrations and has relatively high impacts on modulating the expression of the *SpTrf* gene family, the *SpIL17-9* gene, and the expression of *SpEchinoidin* in cells from most animals. Alternatively, rSpTrf-E2-4, which fails to show saturation binding to phagocytes, has lower or no significant impact on modulating gene expression. This suggests that if the putative SpTrf binding site(s) on a cell reach saturation as with rSpTrf-C1 *in vitro*, the associated putative signaling pathway may be strengthened and subsequent modulation of gene expression may be more pronounced. *In vivo*, a high level of soluble natSpTrf proteins may initiate a negative signaling pathway to reduce or maintain low-level expression of immune genes including the *SpTrf* gene family and other pro-inflammatory genes, thereby preventing aberrant or continued immune responsiveness. Other examples of secreted proteins that regulate their own gene expression includes Nodal in sea urchins, which is a transforming growth factor beta (TGFβ) homologue that initiates positive feedback in larval ectodermal cells to maintain expression by signaling through Suppressor of Mothers against Decapentaplegic (SMAD) (78). Some transcription factors also show auto-regulatory activity by binding to their own gene regulatory regions to modulate transcription. In sea urchins, examples include several transcription factors that function in the gene regulatory network (GRN) that controls development from eggs to early larvae (79, 80) and the GRN sub-routine that controls the differentiation of immune cells in larvae (81). Transcription factors with auto-regulatory function have also been described in immune cells in mammals (82, 83) and in plants that control responses to environmental changes (84). The thyroid hormone nuclear receptor with auto-induction during metamorphosis in amphibians has similar functions in insects (85). Together, our results hint at a sophisticated regulatory mechanism in the sea urchin immune system and provide insights into the possible regulatory activities of the natSpTrf proteins and their putative functions for maintaining homeostasis.

## 5 Conclusions

The SpTrf proteins have activities of major importance in the innate immune system in sea urchins. Immune responsiveness of the *SpTrf* genes is based on their striking up-regulation in response to immune challenge (32, 33, 38) and mRNA editing that expands significantly the sequence diversity among the secreted SpTrf proteins (39) Although these proteins share a general structure and are all predicted to be intrinsically disordered (47, 61), the functions described here and integrated with previous findings (41, 46, 48) suggest that the various natSpTrf proteins have unique functions and a broader range of activities than previously considered. The secreted natSpTrf proteins respond to detected immune challenges, function as opsonins, bind specifically to sites on polygonal and small phagocytes, and augment phagocytosis of foreign targets (46, 48). Alternatively, in the absence of foreign targets, soluble natSpTrf proteins bind to phagocyte surfaces, the complex of protein and binding site aggregates, may undergo endocytosis, and initiates a negative signaling pathway that has regulatory functions for immune responses. When functioning in combination, the structural status of the proteins and their opsonization activities may adjust the level of gene expression to the level of the foreign threat. In sea urchins, the multifaceted functions of natSpTrf proteins likely provide robust immunological benefits and regulation of innate immune responses, ultimately supporting effective host protection and survival.

## Data Availability

The original contributions presented in the study are included in the article/**Supplementary Material**. Further inquiries can be directed to the corresponding author.
