# Supplementary material for: Recombinant SpTransformer proteins bind to specific sites on sea urchin phagocytes and modulate SpTransformer gene expression and immune responsiveness
Source: Front Immunol. 2025 Jan 28;15:1496832. doi: 10.3389/fimmu.2024.1496832 (PMC11810900; doi:10.3389/fimmu.2024.1496832)
Supplement: Supplementary file 1 [file DataSheet1.pdf]

Supplementary Material

Recombinant SpTransformer proteins bind to specific binding sites on sea urchin phagocytes and modulate *SpTransformer* gene expression and immune responsiveness

Ryley S. Crow, Leon Grayfer, L. Courtney Smith*

Department of Biological Sciences, George Washington University, Washington DC, United States

Contents

Figure	Page	Title
S1	2	rSpTrf proteins bind to a subset of phagocytes.
S2	3	The capacity of cells to bind rSpTrf-A6 is not correlated with the expression of natSpTrf proteins.
S3	4	The number of cells that produce natSpTrf proteins is not influenced by binding soluble rSpTrf-A6.
S4	5	The flow cytometry gating strategy to identify the coelomocyte populations that bind rSpTrf-A6.
S5	6	rSpTrf-E2-3 binds to both live and fixed coelomocytes.
S6	7	rSpTrf proteins do not bind to insect cells.
S7	8	The on/off rate of rSpTrf-A6 and -E2-3 bound to fixed coelomocytes is too low to measure.

Table	Page	Title
S1	9	Primers used for qRT-PCR

Supplementary Text File	Page	Title
Text S1	10	rSpTrf protein element pattern is not correlated with protein function.
Figure S8	11	rSpTrf protein element patterns do not correlate with the proteins to function as opsonins, bind phagocytes, drive phagocytosis, or modulate immune gene expression.
Figure S9	12	The rSpTrf protein element patterns are not correlated with the capacity to interact with coelomocytes or modulate gene expression.
Table S2	13	The rSpTrf protein element pattern is not correlated with interactions with coelomocytes.
Table S3	13	The rSpTrf protein element pattern is not correlated with modulating immune gene expression.

References 15

Supplementary Figures

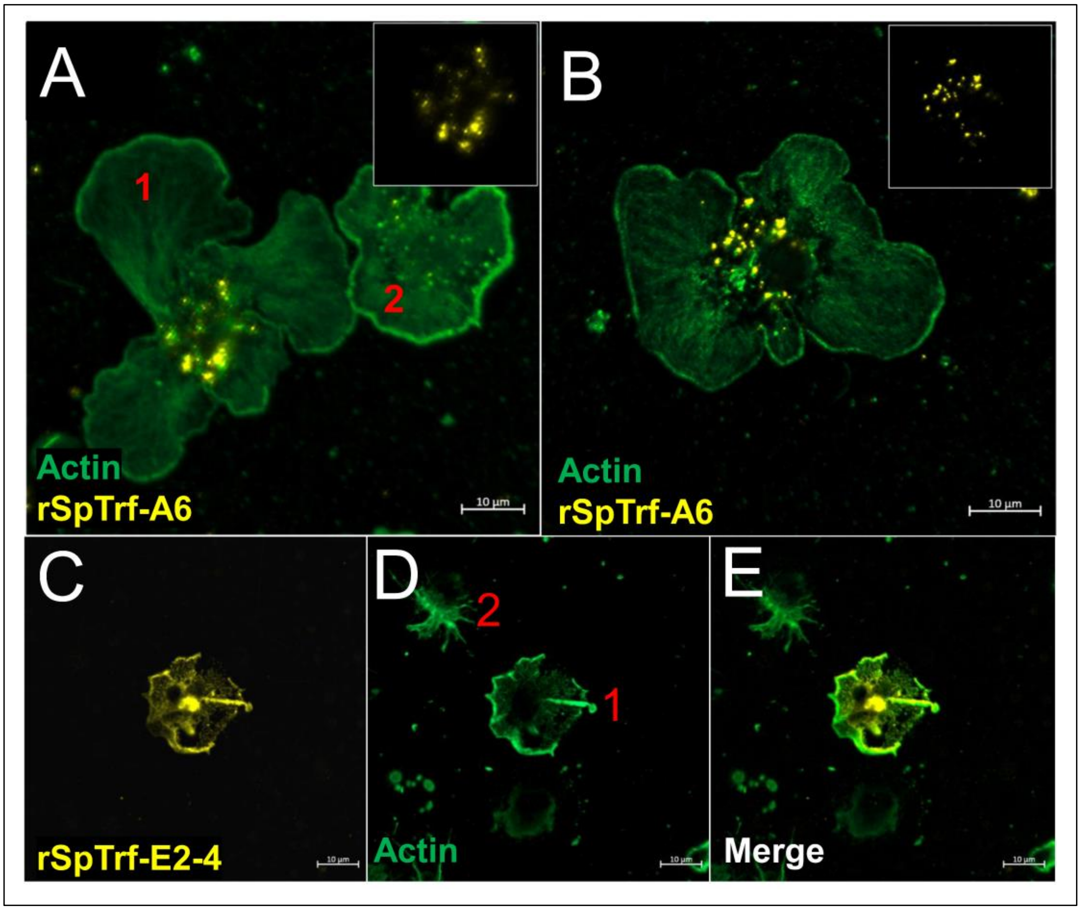

Figure S1
rSpTrf proteins bind to a subset of phagocytes. Both rSpTrf-A6 and -E2-4 bind to a subset of live and fixed polygonal phagocytes, respectively. **(A-B) rSpTrf-A6 binds to a subset of live phagocytes.** **(A)** rSpTrf-A6 binds to polygonal phagocytes in a punctate, perinuclear pattern. Cell 1 shows rSpTrf-A6 binding whereas cell 2 is negative. **(B)** A polygonal cell also shows perinuclear binding of rSpTrf-A6. The nucleus appears as a dark circular region in the center of the cell. The insets in **A** and **B** display the perinuclear rSpTrf-A6 binding without actin labeling. **(C-E) rSpTrf-E2-4 binds to a subset of fixed phagocytes**. Cell 1 binds rSpTrf-E2-4 in a distributed pattern, and cell 2 is negative. Bound rSpTrf-A6 and rSpTrf-E2-4 were detected with ChαV5 followed by GαChIg-405 and cytoskeletal organization was visualized with MαActin followed by GαMIg-488. Cells could not be labeled with DAPI to show the nuclear DNA due to emission overlap with GαChIg-405. Imaging was performed using an LSM 800 confocal microscope (Zeiss) with false color editing applied through the Zeiss image processing software. Scale bars indicate 10 µm.

Figure S2

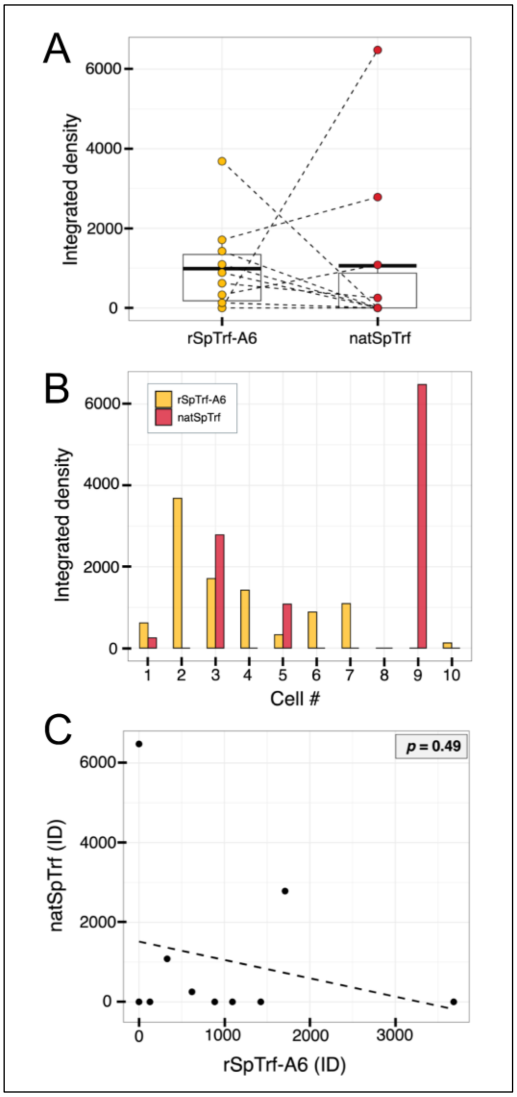

The capacity of cells to bind rSpTrf-A6 is not correlated with the expression of natSpTrf proteins. To assess the relationship between the degree of rSpTrf-A6 binding and natSpTrf expression, fluorescence intensity for both targets was quantified. Coelomocytes were incubated with rSpTrf-A6 in cold aCF for 1 hr and the levels of rSpTrf-A6 binding and natSpTrf expression were evaluated. Data were collected from the confocal cytology files shown as images in Figure 4A in the main paper. The average fluorescence intensity for each phagocyte was measured for ChαV5 followed by GαChIg-405 to detect rSpTrf-A6, and rabbit anti-natSpTrf antibodies (α-66 and α-68) followed by GαRIg-555 to detect natSpTrf using integrated density (ID) that accounted for individual cell sizes. Quantification was performed in Fiji (1), which is an extension of ImageJ. **(A) There is no significant difference in the average fluorescence intensity of bound rSpTrf-A6 compared to natSpTrf expression.** The average ID for rSpTrf-A6 binding and natSpTrf expression in cells 1-10 in Figure 4A show no significant variation as determined by a *t*-test ($p > 0.05$). However, cells with the highest intensity for rSpTrf-A6 binding had very low intensity for natSpTrf expression, and conversely, cells with the highest natSpTrf intensity displayed very low rSpTrf-A6 intensity. Colored dots represent individual cells in Figure 4A, with dotted lines that connect the rSpTrf-A6 (yellow) and natSpTrf (red) fluorescence intensities measured for each cell. **(B) There is no relationship between the fluorescence intensity of rSpTrf-A6 bound to individual cells and their expression of natSpTrf proteins.** The bars represent the ID for bound rSpTrf-A6 (yellow) and natSpTrf expression (red) for cells 1-10 in Figure 4A in the main paper. Analysis of these individual cells does not reveal consistent patterns between the intensities of bound rSpTrf-A6 and natSpTrf expression, in agreement with the results in panel **A**. No trend in natSpTrf intensity was identified among cells that bound rSpTrf-A6 compared to natSpTrf expression at a relatively high (cell 3), low (cell 1), or no natSpTrf expression (cells 2, 4, 6, 7). The failure to identify any correlation indicates that rSpTrf-A6 binding is not dependent on natSpTrf expression, nor is natSpTrf expression dependent on rSpTrf-A6 binding. **(C) There is no correlation between the fluorescence intensity of bound rSpTrf-A6 and the intensity of natSpTrf expression.** When the ID for rSpTrf-A6 is plotted against the ID for natSpTrf expression there is no significant correlation between these values ($p = 0.49$). The dots represent values for individual cells, and the dashed line shows the linear regression line of best fit. There is a slight negative correlation, but the intensity of bound rSpTrf-A6 did not correlate with the intensity of natSpTrf expression. These findings suggest that the amount of natSpTrf produced by a cell does not reflect its ability to bind soluble rSpTrf-A6.

Figure S3

The proportion of cells that produce natSpTrf proteins is not influenced by bound soluble rSpTrf-A6. Although soluble rSpTrf-A6 binds to phagocytes regardless of the level of natSpTrf expression, it was not known whether rSpTrf-A6 influenced the abundance or production of natSpTrf proteins by the cells. To investigate a possible relationship, coelomocytes were incubated with either 1 µg or 0 µg of rSpTrf-A6 in ice cold aCF for 1 hr, and the number of natSpTrf-positive cells was compared between the two treatments. Bound rSpTrf-A6 was detected using ChαV5 followed by GαChIg-405, and the natSpTrf proteins were detected with rabbit anti-natSpTrf antibodies (α-66 and α-68) followed by GαRIg-555. To determine whether responses varied between polygonal and small phagocytes, cell types were identified based on actin cytoskeletal structure, using MαActin followed by GαMIg-488. Discoidal phagocytes were excluded from this analysis, as they do not show specific interactions with rSpTrf-crosslinked beads (2) and have little to no expression of natSpTrf proteins (3, 4). **(A) The proportion of phagocytes that express natSpTrf proteins is not affected by bound soluble rSpTrf-A6.** The overall percentage of phagocytes (both polygonal and small) that express natSpTrf proteins did not differ significantly between cells incubated with 1 µg of rSpTrf-A6 (yellow) and the control cells incubated with 0 µg of protein (teal) (Chi-square test, $p > 0.05$). Although not significant, it is notable that the cells incubated with rSpTrf-A6 had fewer natSpTrf positive cells (46.7%) than cells incubated without protein (64.3%). The lack of significance may be attributed to the low number of observations (1 µg: n = 45, 0 µg: n = 14), as the exclusion of discoidal cells from the analysis reduced the number of observations. **(B) The proportion of polygonal and small phagocytes that express natSpTrf proteins is not affected by bound soluble rSpTrf-A6.** To examine whether there was a phagocyte subtype with activities that correlated natSpTrf expression and bound rSpTrf-A6, the number of natSpTrf positive polygonal and small phagocytes was evaluated within each treatment (see legend). Consistent with overall trends, the number of natSpTrf-positive polygonal and small phagocytes did not differ significantly between treatments (Chi-square test, $p > 0.05$). However, there were fewer natSpTrf-positive polygonal phagocytes (47.8%) when incubated with rSpTrf-A6 compared to the controls incubated without protein (62.5%). Similarly, there were also fewer natSpTrf-positive small phagocytes when incubated with rSpTrf-A6 (45%) compared to the controls incubated without protein (66.7%). Although these differences were not statistically significant, the trends suggested that binding rSpTrf-A6 may reduce natSpTrf production in both polygonal and small phagocytes.

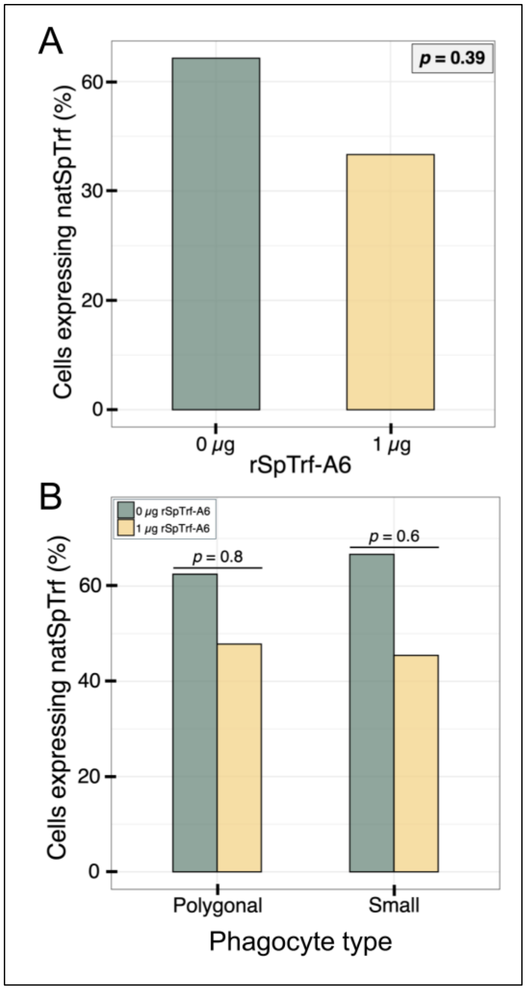

Figure S4

The flow cytometry gating strategy used to identify the coelomocyte populations that bind rSpTrf-A6. Coelomocytes from sea urchin B (SU-B) were incubated with rSpTrf-A6 (A^+–D^+) or without rSpTrf-A6 (A^-–D^-) followed by MαV5-488. **(A) Identification of live cells.** Live coelomocytes were gated by excluding dead cells that took up propidium iodide. **(B) Identification of singlets.** Live cells were examined for linearity in complexity, side scatter area (SSC-A) versus side scatter height (SSC-H) to identify single coelomocytes (red gate) and to exclude clumps of more than one cell. **(C) Identification of coelomocytes.** Debris was excluded from coelomocytes (black polygon gate) based on size (forward scatter; FSC-A) and complexity (SSC-A). **(D) Identification of coelomocyte populations that bind rSpTrf-A6.** Coelomocytes were evaluated for bound rSpTrf-A6 with the 488 nm laser to detect MαV5-488. The specific coelomocyte populations that bound rSpTrf-A6 were differentiated by the 647 nm laser to detect the auto-fluorescence of red spherule cells. The same gating protocol was used for sea urchin A (SU-A), which displayed similar cellular profiles (not shown). For each animal, at least 500,000 events were recorded with a BD Celesta Cell Analyzer flow cytometer. This strategy was based on gates optimized for coelomocytes as reported previously (5-7).

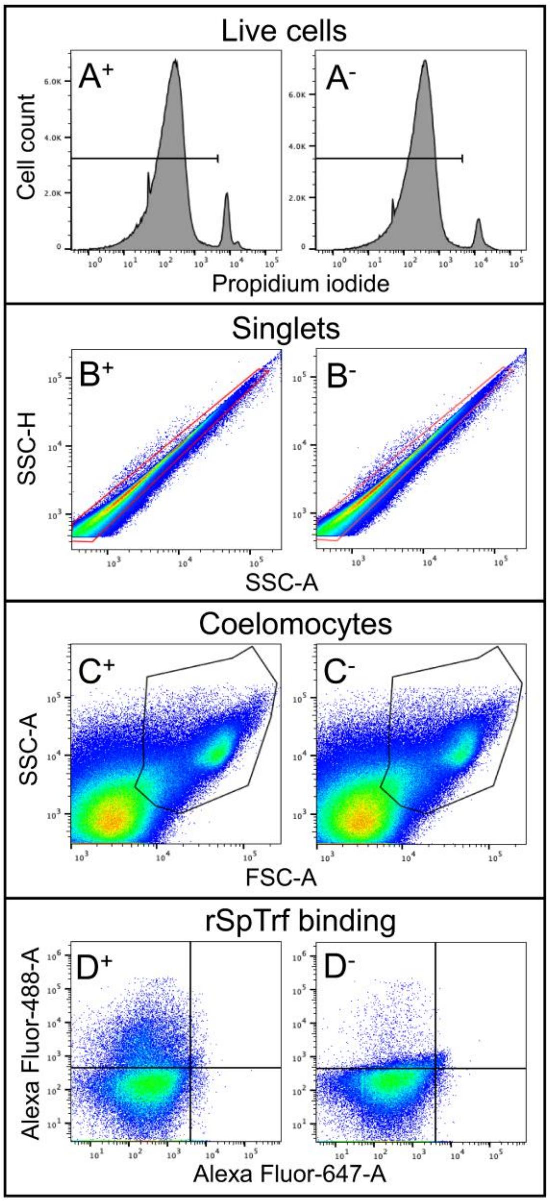

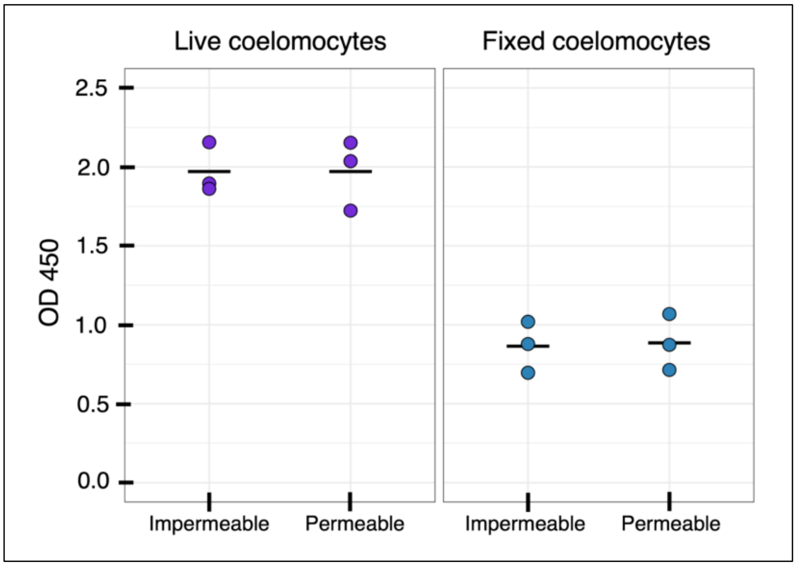

Figure S5
rSpTrf-E2-3 binds to both live and fixed coelomocytes. Live or fixed cells that were either permeabilized or kept impermeable were incubated in triplicate wells with rSpTrf-E2-3 followed by evaluation with MαV5-HRP by In-cell ELISA. Both live and fixed cells bind the protein, and permeability does not alter binding. Black bars indicate the means of three replicates.

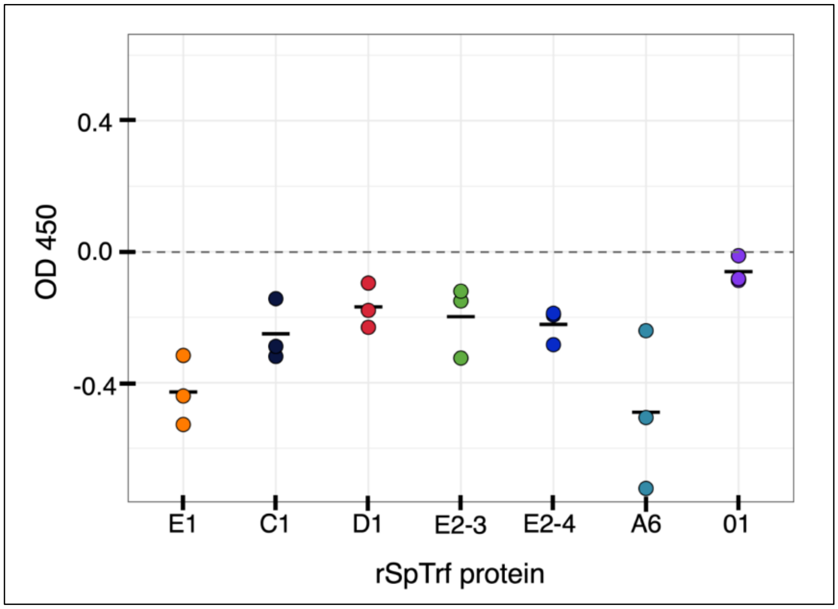

Figure S6
rSpTrf proteins do not bind to insect cells. Each of the rSpTrf proteins (20 nM) were incubated in triplicate wells with fixed Sf9 insect cells and evaluated for binding with MαV5-HRP by In-cell ELISA. The rSpTrf proteins do not bind to insect cells suggesting that these proteins may not bind to cells from eukaryotes other than sea urchin phagocytes.

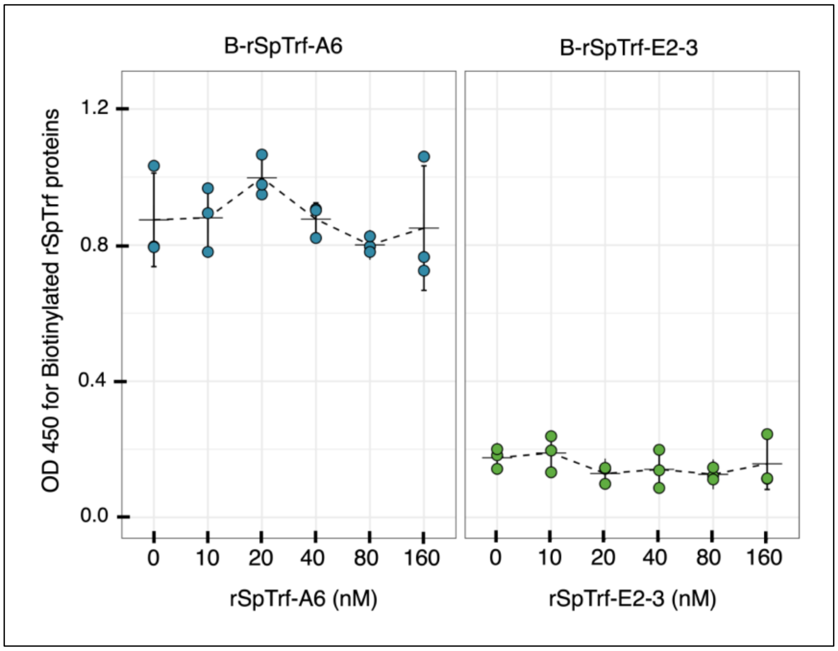

Figure S7

The on/off rate of rSpTrf-A6 and -E2-3 bound to fixed coelomocytes is too low to measure. Coelomocytes from a sea urchin were incubated in triplicate wells with biotinylated (B)-rSpTrf-A6 or B-rSpTrf-E2-3 followed by incubation with increasing concentrations of the same but unlabeled protein. Bound biotinylated proteins were evaluated with streptavidin-HRP by In-cell ELISA. There are no changes in the level of binding by the biotinylated proteins showing that the on/off rate of the rSpTrf proteins after binding to the cell surface is too low to measure suggesting very tight binding. Differences in the binding by B-rSpTrf-A6 and B-rSpTrf-E2-3 correspond to binding results for these two proteins (see Figure 6A, B in in the main paper).

Supplementary Table

Table S1
Primers used for qRT-PCR

**Gene name**; NCBI reference sequence accession number	**Forward (5′−3′)** **Reverse (5′−3′)**
SpL8	F; AGGGCATCGTCAAGGAAATC
XM_790908.5	R; GTTCAGTACGCTTCTTGTACCT
SpEchinoidin	F; GGACTCGATAGAGCTGGGTAT
NM_214671.1	R; CTGTGCAGGGCAGACATATT
***SpTrf* gene family**	F; TGTGGCTGCTCTTGCTATC
Multiple sequences[1]	R; CCTTGTCCTCTCTCTCT
SpIL17-9	F; CAATCAGGAGCCTCTCGAGT
XM_030992590.1	R; GTGCCGTGATTGTATTAACCCT

[1]*SpTrf* primers were designed to amplify >60 genes based on sequences in NCBI.

Supplementary Text File

Text S1
rSpTrf protein element pattern is not correlated with protein function

Introduction

The SpTrf proteins are highly variable that is mostly based on differences in the mosaic arrangement of specific blocks of sequences called elements. Analyses of these sequence differences has suggested that proteins with unique element patterns appear to have diverse functions (2, 8). Therefore, to verify possible links between element pattern and function, differences in function identified in the present study and in (2) were integrated and compared across the various rSpTrf isoforms.

Methods

rSpTrf proteins (rSpTrf-01, -E1, -E2, -C1, -D1, and -A6) were produced using Sf9 insect cells for functional analysis (2). Two proteins with an E2 element pattern, which were produced in separate batches, resulted in rSpTrf-E2-3 that is a monomer, and in rSpTrf-E2-4 that is a dimer. The *p*-values associated with the statistical analyses for the cell surface binding and phagocytosis of beads cross-linked to different rSpTrf proteins, as well as the percentage of cells associated with beads, were compiled from (2). The *p*-values for the cell binding ability of soluble rSpTrf proteins using In-cell ELISA, and changes gene expression in response to binding, based on qPCR, were compiled from the current study. The negative natural logarithm of the *p*-values (-ln(*p*)) established for each rSpTrf protein relative to negative controls (BSA, rCSF-1, or no protein) were used to show functional differences relative to element pattern across the various assays. The calculated values for each protein were summed to produce an overall value and sorted from largest to smallest. This ordering established scores and ranks from best to worst, with the largest value representing the best score, indicating the greatest difference from the control or smallest *p*-value across experiments.

Results

The overall results for the analyses carried out in this study and in (2) do not show correlations between protein element pattern and function. This is consistent with previous hypotheses suggesting that element patterns are not predictive of immune function (2). For example, rSpTrf-E1 and -E2 share many of the same elements (see Figure 1 in the main paper), however, these two proteins do not share functions. rSpTrf-E2-4 is among the most effective proteins for driving phagocytosis of foreign particles, whereas rSpTrf-E1 does not promote phagocytosis (Supplementary Figure S8; Table S2) (2, 9). Although there was no correlation between element pattern and function, some of the rSpTrf proteins had similar rankings for cell interactions and gene modulation (Supplementary Tables S2, S3). For example, rSpTrf-A6 and -01 both ranked highly for cell binding, phagocytosis, and gene regulation (Supplementary Figure S9) ((2), this paper). In contrast, rSpTrf-E1 and -D1 had much lower ranking for many analyses. Similarities in the cellular responses to binding rSpTrf-A6 and -01 reinforced the notion that element pattern alone does not predict function. This is because rSpTrf-A6 is the largest protein with the most elements, while rSpTrf-01 is the smallest with the fewest elements (see Figure 1 in the main paper). Functional studies with nickel-isolated natSpTrf proteins (from coelomic fluid) and rSpTrf proteins from either *E. coli* or SF9 cells, collectively suggest that while the element pattern is

somehow important for function, it cannot be used to predict function based on our current understanding of these proteins.

Figure S8

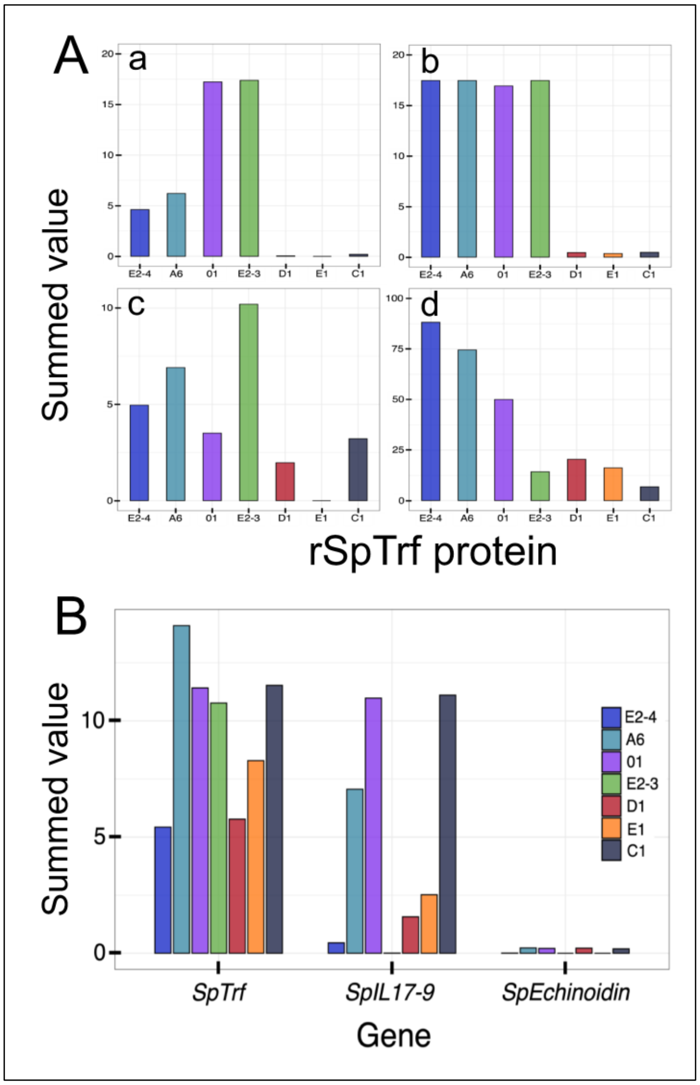

rSpTrf protein element patterns do not correlate with opsonin function, binding phagocytes, augmenting phagocytosis, or modulating immune gene expression. (A) The distinct element patterns of the rSpTrf proteins do not predict interactions with phagocytes. The summed values for individual rSpTrf protein functions, derived from the transformed *p*-values (Supplementary Table S2), are displayed as bar graphs to show functional differences among the rSpTrf proteins cross-linked to beads compared to an irrelevant protein (BSA). Element patterns do not correlate with the capacities of proteins **(a)** to bind phagocyte surfaces or **(b)** to drive phagocytosis of the beads cross-linked to the rSpTrf proteins evaluated by cytology, or **(c)** the percentage of cells associated with at least one bead, and **(d)** the binding capacities of the rSpTrf proteins evaluated by In-cell ELISA. Similarities in functions do not correlate with element patterns of the proteins. **(a-c)** Transformed *p*-values were collected from the results shown in Figures 6 and 7 in Crow et al. (2), and **(d)** transformed *p*-values were collected from the results shown in Figure 6B in the main paper. **(B) The rSpTrf protein element patterns do not correlate with their impact on modulating immune gene expression.** The summed values for individual rSpTrf proteins bound to phagocytes and their impact on gene expression are derived from the transformed *p*-values (Supplementary Table S3) and displayed as a bar graph. *p*-values were collected from the results shown in Figure 8A-C in main paper in which coelomocytes incubated with rSpTrf proteins were compared to coelomocytes incubated without added protein. The bar graph shows no correlation between rSpTrf protein element pattern and the modulation of the *SpTrf* gene family or the *SpIL17-9* and *SpEchinoidin* genes.

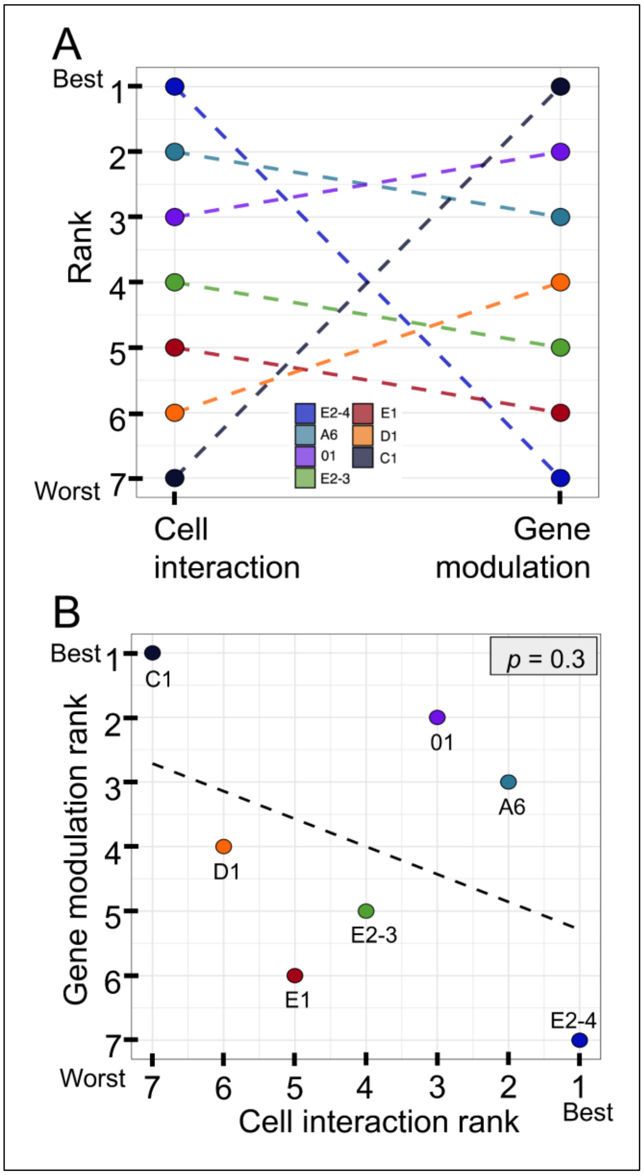

Figure S9

The rSpTrf protein element patterns are not correlated with the capacity to interact with coelomocytes or modulate gene expression. (A) The proteins are ranked based on transformed *p*-values that are indicated as rank 1 to 7 (best to worst; Supplementary Tables S2 and S3). Colored dots indicate the different rSpTrf proteins and dotted lines connect each protein rank for cell interactions and for gene modulation. **(B)** A linear regression (dotted line) of the results indicates that there is no significant correlation among the rSpTrf proteins based on the rank for cell interaction vs. the rank for gene modulation (Pearson's correlation test, $p = 0.3$).

Table S2. The rSpTrf protein element pattern is not correlated with interactions with coelomocytes

Protein	Bead surface binding[1]	Bead phagocytosis[1]	% with beads[2]	Soluble surface binding[2]	Summed value	Rank[3]
E2-4	4.61	17.47	4.96	88.19	115.22	1 Best
A6	6.21	17.47	6.91	74.53	105.11	2 Best
01	17.23	16.94	3.51	50.02	87.69	3 Intermediate 1
E2-3	17.39	17.47	10.20	14.26	59.32	4 Intermediate 1
D1	0.04	0.46	1.97	20.46	22.93	5 Intermediate 2
E1	0.00	0.36	0.00	16.21	16.57	6 Intermediate 2
C1	0.20	0.48	3.22	6.81	10.71	7 Worst

[1]The *p*-values (transformed with -ln(*p*)) for bead surface binding and phagocytosis that were established using a Tukey test, represent the comparisons between the different rSpTrf proteins and BSA. Graphical results are shown in Figures 6A, B and Figure 7 in Crow et al. (2).

[2]The *p*-values (as -ln(*p*)) that were established using a Tukey test from In-cell ELISA reported in the current study (Figure 6B), represent the cell binding abilities of the rSpTrf proteins compared to rCSF-1.

[3]Rank was determined from the summed values of the transformed *p*-values.

Table S3. The rSpTrf protein element pattern is not correlated with modulating immune gene expression[1]

Protein	*SpTrf*	*SpIL17-9*	*SpEchinoidin*	Summed value	Rank
C1	11.53	11.11	0.19	22.83	1 Best
01	11.42	10.98	0.21	22.61	2 Best
A6	14.10	7.06	0.23	21.39	3 Best
E1	8.29	2.52	0.00	10.81	4 Intermediate
E2-3	10.77	0.01	0.00	10.78	5 Intermediate
D1	5.77	1.57	0.22	7.56	6 Worst
E2-4	5.43	0.45	0.01	5.88	7 Worst

[1]The *p*-values established using Dunnett's test were collected from gene expression analysis in the current study (Figure 8A-C). These values represent fold change in gene expression between coelomocytes incubated with different rSpTrf proteins compared to coelomocytes incubated without added protein. Expression analysis for this table employed the values of transformed *p*-values based on -ln(*p*) that were summed to determine rank.

References

1. Schindelin J, Arganda-Carreras I, Frise E, Kaynig V, Longair M, Pietzsch T, et al. Fiji: an open-source platform for biological-image analysis. Nature Methods. 2012;9(7):676-82. doi: 10.1038/nmeth.2019
2. Crow RS, Shaw CG, Grayfer L, Smith LC. Recombinant SpTransformer proteins are functionally diverse for binding and phagocytosis by three subtypes of sea urchin phagocytes. Frontiers in Immunology. 2024;15:1372904. doi: 10.3389/fimmu.2024.1372904
3. Brockton V, Henson JH, Raftos DA, Majeske AJ, Kim YO, Smith LC. Localization and diversity of 185/333 proteins from the purple sea urchin - unexpected protein-size range and protein expression in a new coelomocyte type. Journal of Cell Science. 2008;121(3):339-48. doi: 10.1242/jcs.012096
4. Golconda P, Buckley KM, Reynolds C, Romanello J, Smith LC. The axial organ and the pharynx are sites of hematopoiesis in the sea urchin. Frontiers in Immunology. 2019;10:870. doi: 10.3389/fimmu.2019.00870
5. Smith LC, Hawley TA, Henson JH, Majeske AJ, Oren M, Rosental B. Methods for collection, handling, and analysis of sea urchin coelomocytes. In: Foltz K, Hamdoun A, editors. Methods in Cell Biology; Echinoderms. 150, part A: Elsevier; 2019. p. 391-410. doi: 10.1016/bs.mcb.2018.11.009
6. Yakovenko I, Donnyo A, Ioscovich O, Rosental B, Oren M. The diverse Transformer (Trf) protein family in the sea urchin *Paracentrotus lividus* acts through a collaboration between cellular and humoral immune effector arms. International Journal of Molecular Sciences. 2021;22(1):6639. doi: 10.3390/ijms22136639
7. Barela Hudgell MA, Grayfer L, Smith LC. A flow cytometry based approach to identify distinct coelomocyte subsets of the purple sea urchn, *Strongylocentrotus purpuratus*. Developmental and Comparative Immunology. 2022;130(2022):104352. doi: 10.1016/j.dci.2022.104352
8. Lun CM, Bishop BM, Smith LC. Multitasking immune Sp185/333 protein, rSpTransformer-E1, and its recombinant fragments undergo secondary structural transformation upon binding targets. Journal of Immunology. 2017;198(7):2957-66. doi: 10.4049/jimmunol.1601795
9. Chou H-Y, Lun CM, Smith LC. The SpTransformer proteins from the purple sea urchin opsonize bacteria, augment phagocytosis, and retard bacterial growth. PLoS ONE. 2018;13(5):e0196890. doi: 10.1371/journal.pone.0196890
